# Systematic review of the needs and health-related quality of life domains relevant to people surviving cancer in Europe

**DOI:** 10.1007/s11136-024-03884-w

**Published:** 2025-01-23

**Authors:** Clara Amat-Fernandez, Olatz Garin, Ricardo Luer-Aguila, Yolanda Pardo, Renata Briseño, Catalina Lizano-Barrantes, Leslye Rojas-Concha, Melissa S.Y. Thong, Giovanni Apolone, Cinzia Brunelli, Augusto Caraceni, Norbert Couespel, Nanne Bos, Mogens Groenvold, Stein Kaasa, Gennaro Ciliberto, Claudio Lombardo, Ricardo Pietrobon, Gabriella Pravettoni, Aude Sirven, Hugo Vachon, Alexandra Gilbert, Galina Velikova, Montse Ferrer, the EUonQoL Working Group, Olatz Garin, Leslye Rojas-Concha, Galina Velikova, Massimo Costantini, Madeline Pe, Chiara Marzorati, Antonio Tanzilli, Morten Aagaard Petersen, Aline Machiavelli, Joachim Widder, Helidon Nina, Philip Debruyne, Ivaylo Petrov, Vesna Ramljak, Maria Krini, Tomas Kazda, Helle Pappot, Liina Pääbo, Vahur Valvere, Johanna Mattson, Ann Bredart, Carole Boulec, Mariaalice Borinelli-Franzoi, Ekaterina Kldiashvili, Christian Brandts, Nicole Erickson, Volker Arndt, Olga Balaoura, Horvath Orsolya, Claire Donohoe, Alessandro Rizzo, Andrea Pace, Sandra Lejniece, Audrius Dulskas, Vadim Pogonet, Lonneke van de Poll, Marianne Grønlie Guren, Iwona Ługowska, Maria Litwiniuk, Maria José Bento, Tudor Ciuleanu, Milana Mitrić, Ivica Ratosa, Michal Chovanec, Maria Vieito, Héctor Aguilar, Eva Ruiz, Karin Ahlberg, Eda Tanrikulu Simsek, Mahmut Gumus, Inke Minnée-van Braak, Caitriona Higgins, Laura Pinnavaia, Carina Dantas, Tapani Kalmi, Áurea Martin

**Affiliations:** 1 Health Services Research Group, Hospital del Mar Research Institute, Barcelona, Spain; 2 CIBER en Epidemiología y Salud Pública, CIBERESP, Madrid, Spain; 3 Department of Medicine and Life Sciences, Universitat Pompeu Fabra, Barcelona, Spain; 4 Department of Psychiatry and Legal Medicine, Universitat Autònoma de Barcelona, Barcelona, Spain; 5 Department of Pharmaceutical Care and Clinical Pharmacy, Faculty of Pharmacy, Universidad de Costa Rica, San Jose, Costa Rica; 6 Palliative Care Research Unit, Department of Geriatric and Palliative Medicine GP, Copenhagen University Hospital – Bispebjerg and Frederiksberg, University of Copenhagen, Copenhagen, Denmark; 7 Unit of Cancer Survivorship, Division of Clinical Epidemiology and Aging Research, German Cancer Research Center (DKFZ), Heidelberg, Germany; 8 Scientific Directorate, Fondazione IRCCS Istituto Nazionale Dei Tumori-Milano, Milan, Italy; 9 Dipartimento di Scienze Cliniche e di Comunità, Dipartimento di Eccellenza 2023-2027, Università Degli Studi Di Milano, Milan, Italy; 10 European Cancer Organisation (ECO), Brussels, Belgium; 11 Netherlands Institute for Health Services Research (Nivel), Utrecht, The Netherlands; 12 Department of Public Health, and Bispebjerg/Frederiksberg Hospital, University of Copenhagen, Copenhagen, Denmark; 13 Oslo Universitetssykehus HF, Oslo, Norway; 14 IRCCS National Cancer Institute “Regina Elena” Rome (on behalf of Digital Institute for Cancer Outcomes Research (DIGICORE), Brussels, Belgium), Rome, Italy; 15 Organisation of European Cancer Institutes, Brussels, Belgium; 16 SporeData OÜ, Tallinn, Estonia; 17 Istituto Europeo Di Oncologia IRCCS, Milan, Italy; 18 Unicancer, Paris, France; 19 European Organisation for Research and Treatment of Cancer, Brussels, Belgium; 20 Leeds Institute of Medical Research at St James’s, University of Leeds, Leeds, UK; 21 Leeds Teaching Hospitals NHS Trust, Leeds, UK; 22 Health Service Research Group, Hospital del Mar Research Institute, Barcelona Biomedical Research Park, office 144, 88 Doctor Aiguader street, 08003 Barcelona, Spain; 23 Organization of European Cancer Institutes, Brussels, Belgium

**Keywords:** Quality of life, Cancer survivors, Systematic review, Qualitative research

## Abstract

**Purpose:**

To systematically review qualitative studies on outcomes, needs, experiences, preferences, concerns and health-related quality of life (HRQoL) of people surviving cancer in Europe in the last decade.

**Methods:**

Protocol registered (https://www.crd.york.ac.uk/PROSPERO, ID575065). Inclusion criteria: studies with qualitative methods, constructs related to HRQoL, and adults surviving cancer in Europe. The search was conducted in PubMed and Scopus since 2013. Abstracts and full text were revised, data extracted and study risk of bias assessed independently by two researchers. The primary outcomes were the themes arising from each study. A thematic analysis stratified according to the study objective was undertaken by grouping themes into categories.

**Results:**

Of 18,256 articles identified, 43 fulfilled the inclusion criteria: 16 studies with a generic objective and 27 with specific objectives. Seven categories (57 themes) emerged from the studies with a generic focus: Clinical Management (n = 16), Symptoms and Physical Function (n = 5), Psychological Function (n = 21), Social Function (n = 18), HRQoL (n = 3), Life Disruption (n = 6), and Individual Factors (n = 1). The 12 studies focused on treatment and care experiences stand out among those with specific objectives, with most themes fitting into the same seven categories.

**Conclusions:**

Results clearly showed the predominance of the social and psychological function domains over physical domains among people surviving cancer, additionally identifying specific needs in clinical management, such as information and communication, and relationship with and support from professionals. Therefore, these aspects should be incorporated into the evaluation of patient-centred initiatives for people surviving cancer. Limitations: only two databases were searched, and most European countries were not represented.

**Supplementary Information:**

The online version contains supplementary material available at 10.1007/s11136-024-03884-w.

## Introduction

The number of people surviving cancer is rising worldwide, driven by advances in early detection and treatment and by the aging of the world’s population. In 2020, almost 24 million people (5% of the population) were estimated to be alive after a cancer diagnosis in Europe [1]. In the United States of America, more than 18 million people with a history of cancer were alive in 2022 [2]. For the majority of this population, life after cancer presents lasting challenges [3]: late effects occurring months or years after treatment ends, combined with long-term effects of cancer that impact survivors’ health-related quality of life (HRQoL).

The first patient-reported outcome measure (PROM) designed for measuring HRQoL in cancer survivors was published in 1995 [4] and, since then, five more instruments for survivors of any cancer diagnoses have been developed, three in the 2000s [5–7] and two more in 2014 [8, 9], mainly to be used in research [10]. Nowadays, technology allows for a larger use of PROMs with a considerably lower administration burden [11]. However, their limited adoption in routine care might be related to the content of the existing instruments [12, 13], which may not consider the evolving needs of cancer survivors nor PROMs’ applications beyond research, such as monitoring patients’ clinical management or health services’ quality.

The irruption in the last decade of new treatments such as immunotherapy, targeted therapy or minimally invasive surgeries have changed the experience of people with, and surviving, cancer [14]. To understand the current situation of this population, there has been an increase in the number of qualitative studies and systematic reviews of such studies [15] for identifying their emerging needs, concerns and worries. It is essential to take into account the evidence provided by this qualitative research in the development of new PROMs.

A meta-review of qualitative research on adult cancer survivors identified 60 systematic reviews published between 1998 and 2018 [15], most of which focused on specific tumour location populations. Replicating its search strategy up to July 2024, we identified more than 80 additional systematic reviews published in the last 6 years, half specific for tumour location population and the remainder specific for the construct explored, the most frequent being: unmet needs [16–20], return to work [21–25], psychological well-being [26–28], sexual/reproductive health [29–31], and fear of recurrence [32–34]. Despite the exponential increase of systematic reviews published in the last years, none covered all HRQoL constructs that are important for cancer survivors, without restricting to a specific tumour location population or construct of interest. Thus, a comprehensive summary of qualitative research that identifies the most relevant issues related to quality of life for people surviving cancer nowadays is lacking.

Improving cancer patients’ and survivors’ HRQoL is one of the key action areas of Europe’s Beating Cancer Plan for 2021–2023 [35] and Mission Cancer [36]. The universal health coverage in European countries guarantees access to diagnosis and treatment, thus making the trajectory of people with cancer different from other world regions. Nevertheless, geographic variation in oncological indicators is high even among European countries; for example, mammography screening in women aged 50–69 within the past two years ranged from 83% in Denmark to 30% in Hungary in 2021 [37].

Therefore, our aim was to systematically review qualitative studies focused on disease-related outcomes, needs, experiences, preferences, concerns and quality of life of people surviving cancer in Europe published in the last decade. This review was performed within the context of the European project EUonQoL [38], which aims at developing a new PROM (EUonQoL toolkit) to assess HRQoL across cancer patients and survivors in Europe. The synthesis of the evidence from this review has provided valuable evidence for the development of the EUonQoL toolkit. It also offers recommendations for other HRQoL instruments designed for people surviving cancer, particularly for identifying domains usually unmet in the traditional HRQoL conceptual models.

## Methods

The protocol of this systematic review and its reporting follow the Preferred Reporting Items for Systematic Reviews and Meta-Analyses (PRISMA) [39] and is under review in the International Prospective Register of Systematic Reviews database (ID 575065 in https://www.crd.york.ac.uk/PROSPERO).

### Eligibility criteria

We considered as inclusion criteria: studies with qualitative methods (including also mixed method approaches) focused on disease-related outcomes, needs, preferences, concerns, worries, or quality of life; in samples of people surviving cancer (disease-free without evidence of active cancer, and at least one year off active treatment—except for long-term adjuvant hormonotherapy); performed in the 27 countries belonging to the European Union (EU), the United Kingdom (UK), and 11 associated countries (Supplementary Table 1 contains the complete list of 39 countries). Only peer-reviewed articles published in European languages were considered for this review.

Studies were excluded if samples were composed of children, adolescents and young adults (diagnosed of cancer at the upper age limit of 39 years [40]); cancer patients in active treatment or in palliative care; very specific populations (e.g., rare tumours, second malignancy, infrequent treatments); patients with multimorbidity (with and without cancer); partners, caregivers or health professionals; aiming to explore tumour location-specific dimensions; or if data was collected prior to 2013 to focus on people surviving cancer being managed in the last decade.

### Information sources

The search was conducted initially in the MEDLINE bibliographic databases (specifically PubMed) on March 6th, 2023, and updated until July 8th, 2024 in MEDLINE and Scopus databases.

### Search strategy

The search strategy in PubMed, which included both MeSH and text word terms, had 4 sections: one focused on the type of population (survivors, patients under treatment or palliative patients), a second one on the pathology (neoplasm), a third section regarding the constructs of interest (related to quality of life), and a last one referring specifically to relevant issues. The search was limited to publications in European languages since 2013. Supplementary Table 2 shows search strategies for PubMed and Scopus.

Several search strategies were tested and the final decision was made based on two simple sensitivity analysis approaches: results including well-known studies in the area of interest; and comparison of the potentially included articles using a strategy restricted to subheadings of MeSH terms, versus a wider strategy (non-restricted to subheadings). The latter strategy included almost 16% more articles than the restricted one, thus no subheadings were applied to the MeSH terms.

Although the original search was larger in scope, covering all patients in the cancer continuum addressed in the EUonQoL project, results presented here are restricted to people surviving cancer, due to the major differences between their experiences and the ones from patients in active treatment and palliative care.

### Selection process

All steps of the screening process were performed with Covidence™ software (www.covidence.org), and its automatic function to remove duplicates was used. Each title and abstract was reviewed independently by two out of the six researchers (CA, OG, MF, CLB, RL, LRC) after a pilot test to standardize criteria. Disagreements in all phases were resolved through discussion with the participation of third-party reviewers.

### Data collection process

For each study, full text review and data extraction was carried out independently by two researchers (CA, RL, OG, YP, RB, CLB, MT, LRC), completing an ad-hoc data extraction form created for this review. A third reviewer cross-checked the data extraction tables for accuracy and completeness.

### Data items

Information extracted included:

1. *Study characteristics*—author, aim of the study, study design, country and year of data collection, recruitment methodology, theoretical approach, qualitative method.
2. *Sample characteristics*—tumour location, sample size, age, sex, time since treatment, operation, chemotherapy, or diagnosis.
3. *Reporting of information*—use of guidelines for qualitative research, saturation of information, themes, subthemes and verbatims.

### Study risk of bias

To assess the risk of bias of the included studies, we used the Specialist Unit for Review Evidence Qualitative Studies Critical Appraisal (SURE) checklist [41]. It is composed of 10 questions that should be answered as ‘Yes’, ‘Can’t tell’, or ‘No’, about: (1) the study addressing a clearly focused question/hypothesis, (2) the choice of qualitative method being appropriate, (3) the sampling strategy being described and justified, (4) the method of data collection being well described, (5) the relationship between the researchers and participants being explored, (6) ethical issues being explicitly discussed, (7) the data analysis/interpretation process being described and justified, (8) the findings being credible, (9) any sponsorship or conflict of interest being reported, and (10) the study identifying any limitations and the conclusions in the full text matched the ones in the abstract. This assessment was performed by the same researchers as the data extraction. Because SURE does not have a global score, we qualified those studies assessed as ‘No’ in three or more of the ten items as ‘poor quality’.

### Outcomes

The themes and subthemes arising from each study included (and the specific verbatims when necessary) were the primary outcome. The themes and subthemes were extracted literally from each article.

### Synthesis methods

Wilson & Cleary’s framework on HRQoL [42], which is currently the most applied theoretical model of HRQoL [43], was followed. The thematic analysis was conducted by a panel of researchers. A deductive phase was implemented in the first stage of synthesis to place the themes and subthemes into categories within the domains of the Wilson and Clearly framework. In a second stage, an inductive and iterative approach was followed to allow subcategories to emerge from their content to address the review question until agreement was reached among the panel of researchers.

### Reporting bias assessment

Sensitivity analysis was planned by replicating the analysis only on studies of good quality (less than three of the ten items in the SURE checklist with a negative qualification). Furthermore, the analysis was stratified according to the objective of the qualitative study into a main thematic analysis centred in the results from those studies with a generic focus, and a secondary thematic analysis with studies with specific focus. This strategy was applied in order to avoid the overrepresentation in the synthesis of results from studies aiming to explore specific constructs.

## Results

### Study selection

**Fig. 1:**
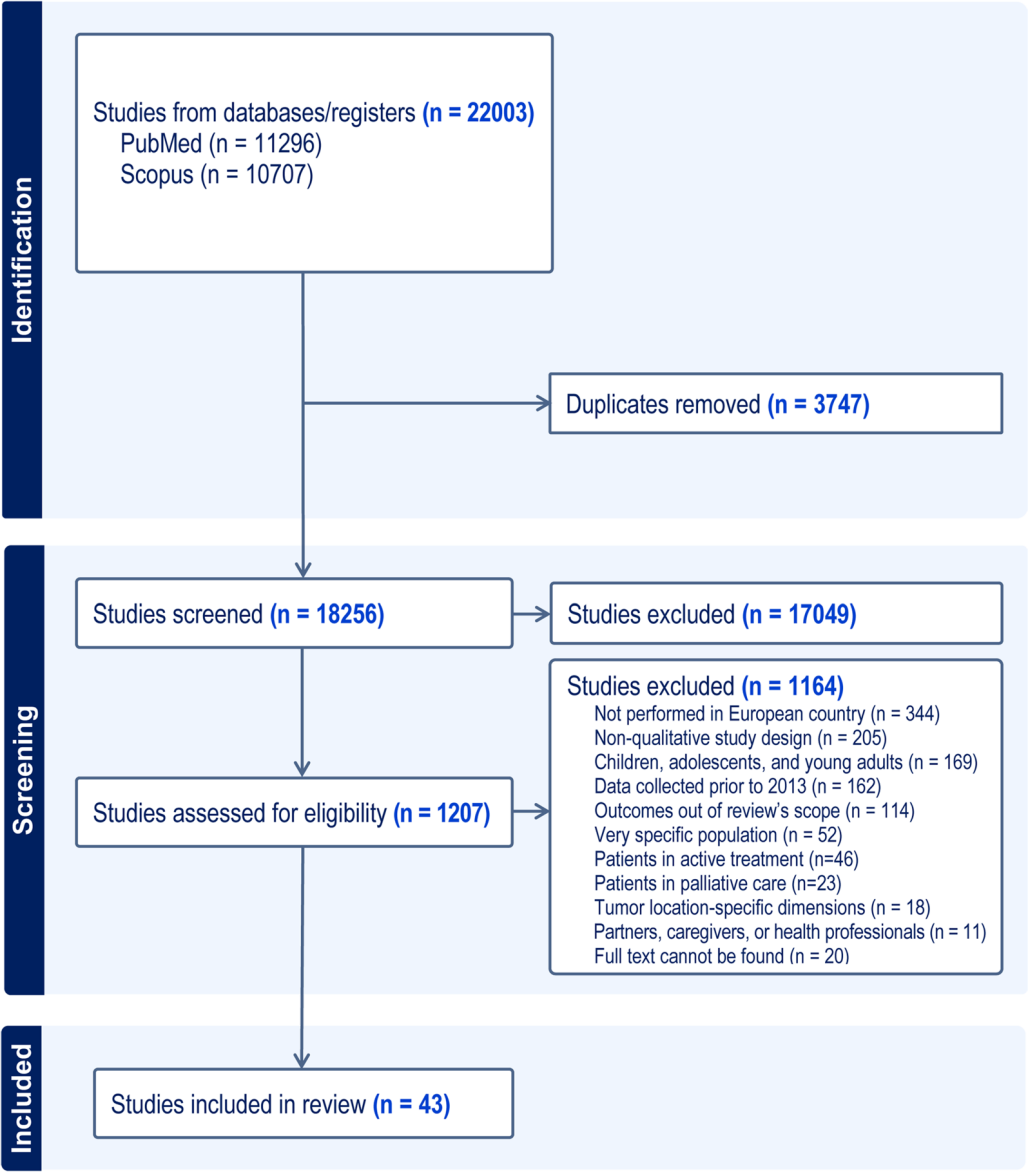
Selection process overview—PRISMA flow-chart

A total of 18,256 articles were identified across PubMed and Scopus. Detailed information of the study selection process is described in the PRISMA flowchart (Fig. 1). After screening titles and abstracts, a complete full-text review of 1207 manuscripts was carried out. The most frequent reasons for exclusion of studies at this stage were: not performed in European countries (30%), non-qualitative study design (18%), children, adolescents and young adults (15%), data collected prior to 2013 (14%), and outcomes out of this review’s scope (10%). Finally, 43 qualitative studies on people surviving cancer fulfilled the inclusion criteria and entered the following phase for data extraction.

### Study characteristics

A summary of the studies’ characteristics is shown in Table 1. The countries in which more studies had been conducted were the UK (n = 14), the Netherlands (n = 6), Norway (n = 5), France (n = 3), Ireland (n = 3), and Turkey (n = 3). The data collection method most often used was semi-structured interviews (n = 26), while 2 studies applied nonstandard approaches: one performed content analysis of blog data and the other audio-recorded follow-up visits. Seven studies included patients with various tumour locations and, among those with specific tumour location samples, the most frequent were breast (8 studies), prostate (7 studies), and colorectal (7 studies). Women represented around 50% of the sample in half of the non-gender related cancer studies. A substantial portion of the studies aimed to explore disease-related outcomes, experiences, needs, concerns, preferences and quality of life of people surviving cancer in general [44–59], but 27 studies focused on more specific objectives: 12 on experience with treatment, services and self-management [60–71]; 3 on late effects [72–74], 3 on working situation [75–77], 3 on psychological distress [78–80], and 6 on other subjects (existential experiences, attitudes and awareness, common language of cancer, fear of recurrence, treatment decision making, and transition to long-term survivorship) [81–86].

**Table 1:** Characteristics of the included studies

	Number of studies
Country	
United Kingdom	14
the Netherlands	6
Norway	5
France	3
Ireland	3
Turkey	3
Denmark	2
Sweden	2
Germany	1
Italy	1
Israel	1
Multiple countries	2
Year of publication	
2013–2015	6
2016–2018	13
2019–2021	9
2022–2024	15
Qualitative approach	
Semi-structured interviews	26
In-depth interviews	5
Focus groups	6
More than one	4
Others	2
Tumour location	
Multiple locations	7
Breast	8
Prostate	7
Colorectal	7
Ovarian, endometrial or cervical	3
Head & neck	3
Brain	2
Melanoma	2
Lung	1
Testicular	1
Non-Hodgkin’s Lymphoma	1
Gender (% of women)*	
< 25%	3
25–49%	8
50–74%	11
≥ 75	1
Aim focus	
Experiences, needs, Quality of life	16
Treatment, services, self-management	12
Late effects	3
Working situation	3
Psychological distress	3
Other	6

*Excluding gender-dependent tumours

### Risk of bias in studies

Supplementary Table 3 shows the quality of the included studies, assessed following the SURE checklist [41]. All 43 studies addressed a clearly focused question/hypothesis (100%), and most of them also explicitly discussed ethical issues (95.3%), made an appropriate choice of the qualitative methodology used for their aim (93%), presented credible findings (93%), reported whether they had any conflict of interest (90.7%), described and justified the data analysis and interpretation (86%), correctly identified the study’s limitations (88.4%), and clearly described their sampling strategy (72.1%) and the method used for data collection (65.1%). The ‘relationship between the researcher and the participant’ item frequently downgraded the studies’ quality, being reported in only 23.3% of the studies.

Three studies were assessed as poor quality: one focused on treatment [62], one on common language of cancer [81], and one on existential experiences [83]. As none of them were within the studies with general objectives, the sensitivity analysis was not needed in the main thematic analysis.

### Results of individual studies

Table 2 shows the characteristics of studies and the identified themes. The qualitative studies with a generic focus on survivors’ outcomes, experiences, needs, concerns, preferences and quality of life were mostly published in 2020–2024, with sample sizes ranging from 6 to 196 participants. In total, 57 themes emerged from these 16 studies with the number of themes at study level ranging from 1 to 6.

**Table 2:** Characteristics and results of studies categorized according to their objectives

Study (author, year, country, tumour location)	Design and methods	Participants (% women, age, time since T, O, C, and D)	Aim of study	Themes (as reported in the study)
Generic focus on disease related outcomes, experiences, preferences, needs, concerns, QoL				
Appleton 2013 UK [44] Colorectal (n = 13)	Phenomenology Semi-structured interview	38.5% women Age:: 45-84y T: 6 m–5y	To explore in-depth the lived experience of colorectal cancer survivors	⋅ Partnership with the multidisciplinary team ⋅ Enablers ⋅ Self beyond cancer
Aunan 2021 Norway [45] Prostate (n = 16)	Not reported Focus group	0% women Age: 55-80y Not reported	To explore and analyse prostate cancer survivors’ experiences and critical reflections of information received during their cancer trajectory	⋅ ‘To be met with interest and support’ ⋅ ‘Enough knowledge to understand what was happening’ ⋅ ‘A plan to build the new life on’
den Bakker 2018 the Netherlands[46] Colorectal (n = 22)	Phenomenology Focus group	50% women Age: 35-76y (M = 65y) O: 8–22 m C: 2–17 m	To gather participants’ experiences with their full recovery […] and […] needs…	⋅ After chemotherapy phase
Drury 2022 Ireland [47] Colorectal (n = 22)	Phenomenology Semi-structured interview Mixed-methods	45.5% women Age: 47-78y D: 1-5y	To explore the prevalence of colorectal cancer survivorship issues and their impact on survivors’ quality of life	⋅ The Vestiges of CC: Loss and Control ⋅ The Shadow of CC: Fear and Vigilance ⋅ Living Beyond CC: Impact and Benefit
Ghirotto 2023 Italy [48] Laryngeal (n = 19)	Phenomenology In depth interview	21% women Age: 41-90y, $\overline{X }=$ 66.3y C: 3 m- > 5y	To explore how persons who had undergone total laryngectomy perceived themselves as cancer survivors in the follow-up phase	⋅ Accepting a life with the “without” to survive ⋅ Feeling unpleasant emotions ⋅ Getting the hang of communication again ⋅ Reclaiming one’s role
Jakobsen [49] Norway [49] Breast (n = 11)	Phenomenology Semi-structured interview	100% women Age: 48-74y Not reported	To describe the everyday life in breast cancer survivors experiencing challenges	⋅ Bodily and mental loneliness ⋅ New centre of gravity in everyday life
Kamminga 50 the Netherlands [50] Melanoma (n = 20)	Grounded Theory Focus Semi-structured interview	0% women Age: 32-83y Not reported	To gain an in-depth understanding of metastatic melanoma survivors’ experiences of resuming life after immune checkpoint inhibitors and their associated survivorship care needs	⋅ Dealing with a switch in prognosis ⋅ Challenges to proceed with life as prior to metastatic cancer ⋅ Finding a new balance ⋅ Needs regarding (medical) information and care ⋅ Need for broader supportive care
Levin-Dagan 2024 Israel [51] Multiple locations (n = 24)	Not reported Focus group	0% women Age: 25-76y, $\overline{X }=$ 51.41y D: 11 m-16y	To focus solely on men who survived various types of cancer and to explore how they perceived and described the post-cancer changes in their lives	⋅ Negative manifestations of cancer survivorship ⋅ Changes in perceptions of life ⋅ Changes in perceptions of self ⋅ Putting changes into action
Mahmood 2024 UK [52] Multiple locations (n = 18)	Phenomenology In-depth interviews	44.4% women Age: 44-76y T: < 5y	To explore cancer survivorship in urban people living with cancer who have completed primary treatment	⋅ Problematic events ⋅ Pre-existing factors ⋅ Environmental factors ⋅ Personal factors ⋅ Healthcare factor
Millet 2022 UK [53] Cervical (n = 21)	Not reported Semi-structured interview	100% women Age: 28-55y; $\overline{X }=$ 37y T: < 2–10y	To explore the recovery experience in the short and long term […] from a biopsychosocial perspective	⋅ Treatment as a paradox ⋅ Emotional fluctuations ⋅ Adversarial growth
Piil 2022 Denmark [54] Brain (n = 13)	Pragmatic paradigm Semi-structured interview Mixed-methods	54% women Age: 31-71y; M = 56y D: > 3.5-12y	To address perspectives on the daily life experiences of long-term survivors with high grade glioma and their caregivers	⋅ Searching for meaningful activities ⋅ Selecting information that enhances self-management strategies ⋅ Protection for safety reasons
Puppo 2020 France [55] Ovarian (n = 16)	Not reported Semi-structured interview	100% women Age: 25-74y; $\overline{X }=$ 63,8y D: 5-16y	To explore how ovarian cancer survivors give meaning to their cancer experience and how the latter has an impact on their quality of life	⋅ Body and physical issues ⋅ The impact of cancer experience on social life ⋅ The impact of cancer experience on perception of life
Samsøe 2022 Denmark [56] Head & Neck (n = 6)	Hermeneutical tradition Semi-structured interview	0% women Age: 51-66y Not reported	To gain insight into men’s experience concerning the QoL one year after completing radiation therapy…	⋅ Overwhelmed by information ⋅ Talking about mental well-being ⋅ Transitions—Cured but not healed ⋅ The fine details to quality of life
Stuhlfauth 2018 Norway [57] Colorectal (n = 9)	Biopsychosocial model Semi-structured interview	56% women Age: 51-73y O: 8-18 m	To gain insight into how persons who have undergone surgery for colon cancer experience changes in their everyday life in general and in their sexual life in particular	⋅ Changes in the body ⋅ Changes in social life ⋅ Changed relationships with partners ⋅ Reviewing one´s perspectives of life-influenced on coping strategies
van Ee 2018 the Netherlands [58] Prostate (n = 22)	Phenomenology Semi-structured interview	0% women Age: $\overline{X }=$ 74.6y D: 0- > 12y	To gain more insight into the experiences of men 70 years old or older with prostate cancer and the care received from health-care professionals, family members and other informal carers	⋅ Impact of prostate cancer ⋅ Dealing with prostate cancer and treatment ⋅ Involvement of and with others ⋅ Experiences with the professional care and the care trajectory
Zanchetta 2016 France [59] Prostate (n = 196)	Ethnography Blog entries	0% women Not reported	To explore issues of quality of life as reported […] in a public blog; to identify the salient aspects and issues of the experience of living with prostate cancer […] based on textual data from their posted testimonies; and to analyse the ideas […] on quality of life	⋅ Self-identification ⋅ Reactions to experiences ⋅ Impacts on quality of life ⋅ Physical functioning ⋅ Psychological and social role functioning ⋅ PC-treatment-related issues
**EXPERIENCES WITH TREATMENT, SERVICES AND SELF-MANAGEMENT**				
Anderson 2013 UK [60] Colorectal (n = 40)	Not reported Focus group	50% women Age: 27-84y, $\overline{X }=$ 60y T: 1-48 m	To explore perceived patient needs for advice on diet, activity and beliefs about the role of lifestyle for reducing disease recurrence	⋅ Perceived need for advice on diet, physical activity and lifestyle UK ⋅ Beliefs about the role of diet, activity and lifestyle for reducing disease risk in the longer term ⋅ Casual beliefs ⋅ Health maintenance actions ⋅ Patients interest in guidance on diet, activity and lifestyle to reduce disease risk and progression ⋅ What are the preferred formats, timings and routes of delivery for guidance on diet, activity and lifestyle?
Burden 2016 Sweden [61] Colorectal (n = 25)	Phenomenology Semi-structured interview	28% women Age: $\overline{X }=$ 67.7y O: 7–30 m	To explore people’s relationships with food and nutrition throughout their colorectal cancer journey	⋅ Appetite swings ⋅ Emotions on changing physicality ⋅ Weight gain ⋅ Medicalisation of food ⋅ Taking control of symptom management ⋅ Drivers for action
Dunne 2018 Ireland [62] Head & Neck (n = 26)	Not reported Semi-structured interview	30.8% women Age: 77% > 55y D: 8-60 m	To identify survivors’ perceptions of barriers to their active self-management after completing primary treatment for head and neck cancer	⋅ Emotional barriers ⋅ Symptom-related barriers ⋅ Structural barriers ⋅ Self-evaluation barriers
Harrow 2014 UK [63] Breast (n = 30)	Phenomenology Semi-structured interview	100% women Age: n = 2 < 50y; n = 15 50-64y; n = 12 > 64y D: 1-5y	To explore women’s experiences of taking adjuvant endocrine therapy; […] factors which influenced adherence […] information and support they received or desired	⋅ Reasons for taking adjuvant endocrine therapy ⋅ Experiences taking adjuvant endocrine therapy ⋅ Perceptions of and need for support
Koutoukidis 2017 UK [64] Endometrial (n = 16)	Not reported Focus group Semi-structured interview	100% women Age: M = 57.4y T: < 5y	To examine the perceived importance of health behaviours after […] cancer treatment, and the factors influencing adherence to […] and […] method of information delivery	⋅ Defining a healthy life-style ⋅ Factors influencing diet and physical activity ⋅ Needing to search for information
Marshall-McKenna 2023 Greece, Spain, Sweden and UK [65] Multiple locations (n = 7)	Not reported Semi-structured interview Mixed-methods	70% women Age: $\overline{X }$>65y: 71,5y/ $\overline{X }$ 50-64y: 57,2y D: < 1- > 25 m	To evaluate healthcare needs, preferences, and expectations in supportive cancer care as perceived by cancer survivors, family caregivers, and healthcare professionals	⋅ Priorities in life post-treatment ⋅ Health concerns/needs relating to age in survivorship ⋅ Support/information since the end of treatment ⋅ Family support needs ⋅ Concerns due to COVID ⋅ Ideal health services ⋅ Ideal support ⋅ Expectations of health professionals’ actions ⋅ Comfort with technology
Pallin 2022 Ireland [66] Non-Hodgkin’s Lymphoma (n = 8)	Phenomenology In-depth interviews	25% women Age: 56-87y D: 3-14y	To explore the views on self-management and preferences for self-management support among survivors of low-grade non-Hodgkin’s lymphoma and their informal caregivers more than 6 months after completion of systemic anti-cancer therapy	⋅ The chronic nature […] shapes perceptions of self-management ⋅ Social networks enable self-management ⋅ Support and monitoring are needed immediately after the initial treatment phase ends ⋅ Preferred components of self-management
Regnier Denois 2017 France [67] Breast (n = 36)	Phenomenology Focus group In-depth interviews	100% women Age: 21% < 40y; 31% 40-45y: 48% 45-50y T: 6-24 m	To understand the barriers to using supportive care services among breast cancer survivors under the age of 50 and to find out how this can contribute to inequalities	⋅ Lack of awareness of supportive care service ⋅ Limited access to support services and resources ⋅ Barriers stemming from patients’ mental image of supportive care services ⋅ Unmet needs in supportive care services
Seibel 2023 Germany [68] Lung (n = 25)	Not reported Semi-structured interview	52% women Age: 52-58y; $\overline{X }=$ 67y T: 0–11y	To explore the subjective experience of follow-up care and its possible psychosocial effects on everyday life from the perspective of lung cancer survivors and their caregivers	⋅ Ongoing impact of curatively treated cancer in the family system: long-term and late effects ⋅ Meaning of follow-up care ⋅ Psychosocial needs during follow-up care
Stamataki 2015 UK [69] Melanoma (n = 15)	Phenomenology Semi-structured interview	53.3% women Age: 27-78y; $\overline{X }=$ 52y D: > 3 m-5y	To explore the impact of melanoma diagnosis on the supportive care needs of patients with cutaneous melanoma	⋅ Emotional effects ⋅ Effects on relationships ⋅ Functional effects ⋅ Health care system and information needs
Voigt 2024 the Netherlands [70] Colorectal (n = 19)	Not reported Focus group Semi-structured interview Mixed methods	47% women $Age:\overline{X }=$ 65y O: 0-9y	To assess the needs of colorectal cancer patients regarding their follow-up care	⋅ Cancer and life ⋅ The healthcare system ⋅ CEA-value ⋅ Quality of life questionnaires ⋅ Information provisions ⋅ Remaining platform issue
Wollersheim 2021 the Netherlands [71] Prostate (n = 32)	Not reported Recording of visits	0% women Age: 63y (Mn) Not reported	To investigate the supportive care and information needs of prostate cancer survivors during routine follow-up care	⋅ Health system and information ⋅ Physical and daily living ⋅ Psychological ⋅ Sexuality
**LATE EFFECTS**				
Treanor 2016 UK [72] Multiple locations (n = 7)	Phenomenology Semi-structured interview	56.3% women Age: 39-75y D: 3–21y	To investigate the nature and onset of late effects experienced by survivors and the manner in which late effects have affected their lives	⋅ Onset and nature ⋅ Management ⋅ Impact of late effects ⋅ Personal disposition ⋅ Peer comparisons ⋅ Sense making
Trusson 2016 UK [73] Breast (n = 24)	Not reported In-depth interviews	100% women Age: 42-80y; $\overline{X }=$ 51y T: 6 m–29y	In depth consideration of ongoing disruptions to identities, bodies and relationships, from diagnosis of breast cancer to the end of treatment, and well beyond	⋅ Biographical disruption and liminality ⋅ Fear of recurrence ⋅ Embodied reminders ⋅ Relationships ⋅ Changes in outlook
Wennick 2017 Sweden [74] Prostate (n = 19)	Not reported Semi-structured interview	0% women Age: 59-65y; $\overline{X }=$ 60,7y; M = 62y O: 12-18 m	To illuminate how men < 65 years of age experience their everyday life […] after a radical prostatectomy […], side effects…	⋅ Paying a price for survival ⋅ Feeling sidestepped ⋅ Living with death lurking around the corner
**WORKING SITUATION**				
Liaset 2018 Norway [75] Brain (n = 4)	Not reported In-depth interviews	Gender: Not reported Age: 30-59y Not reported	To explore individual experience after undergoing treatment for brain cancer and the return-to-work process	⋅ Back at work 100% after a couple of months ⋅ To be a minus ⋅ Adjustments of work tasks is everything ⋅ Those who are closest have a lot to say – hard without
Şengün İnan 2020 Turkey [76] Breast (n = 12)	Phenomenology Semi-structured interview	100% women Age: 33-58y; $\overline{X }=$ 48y T: 14-36 m	To explore experiences of Turkish breast cancer survivors about returning or continuing to work	⋅ Decision making for returning to work ⋅ Difficulties in work life ⋅ Sources of motivation for continuation of work ⋅ Benefits of returning to work
Torp 2020 Norway [77] Multiple locations (n = 7)	Not reported Semi-structured interview	85.7% women Age: Not reported	To explore how self-employed people experience their working situation during and after cancer treatment	⋅ Entrepreneurship and engagement ⋅ Cancer treatment and late effects ⋅ Business related worries ⋅ Shame ⋅ Support
**PSYCHOLOGICAL DISTRESS**				
Matheson 2020 UK [78] Prostate (n = 28)	Phenomenology Semi-structured interview	0% women Age: 46-87y; $\overline{X }=$ 65.9y Not reported	To explore the experiences of men identified as having psychological distress, drawn from the total sample of interviewed men […]	⋅ Perceptions of loss ⋅ Maladaptive strategies for coping with distress
Reynolds-Cowie 2021 UK [79] Multiple locations (n = 27)	Phenomenology Focus group	59% women Age: $\overline{X }=$ 62y T: > 1 m	To investigate the impact of insomnia on cancer survivors’ lives; to provide insight into the strategies used […] to self-manage insomnia; to explore the attention given to sleep difficulties […]; and to consider the availability of support or interventions […]	⋅ I don’t feel like myself ⋅ Planning life around something uncontrollable ⋅ My body hurts ⋅ My brain is not functioning ⋅ It’s more than just not sharing a bed ⋅ Worry
Şengün İnan 2023 Turkey [80] Breast (n = 18)	Phenomenology Semi-structured interview	100% women Age: 32-70y; $\overline{X }=$ 49y T: 4-28 m	To explore the unmet supportive care needs of breast cancer survivors who experience psychological distress	⋅ Sources of psychological distress ⋅ Unmet support needs ⋅ Barriers to support
**OTHER**				
Appleton 2014 UK [81] Not reported (n = 18)	Phenomenology Focus group	61% women Age: 45-85y Not reported	To gain an insight into how survivors experience the common language and metaphor of cancer	⋅ Journey ⋅ Survivor / Patient ⋅ Normality ⋅ Managing identity ⋅ Managing emotions
Deery 2023 Ireland, UK [82] Breast (n = 8)	Not reported Semi-structured interview	100% women Age: 45-64y Remission > 2 y	To investigate […] attitudes towards their health post‐treatment, […] co‐morbidities […] support systems available	⋅ Health and rehabilitation post‐treatment ⋅ Access to support services in survivorship
Lagerdahl 2014 UK [83] Multiple locations (n = 8)	Not reported Semi-structured interview	62.5% women Age: 43-62y; $\overline{X }=$ 55y T: 2-12 m	To explore the existential experiences of patients who have undergone treatment with curative intent for a range of cancers, and are considered to be in complete remission	⋅ Death anxiety ⋅ Freedom ⋅ Isolation ⋅ Meaning
Şengün İnan 2019 Turkey [84] Breast (n = 12)	Phenomenology Semi-structured interview	100% women Age: 33-70y T: 7-23 m	To explore Turkish breast cancer survivors’ experiences related to fear of recurrence	⋅ Quality of Fear ⋅ Triggers ⋅ Effects on Life ⋅ Coping
Wagland 2019 UK [85] Prostate (n = 97)	Phenomenology Semi-structured interview Mixed methods	0% women Age: 48-87y; $\overline{X }=$ 66y D: 18-42 m	To explore the experience of treatment decision making amongst men diagnosed with stage I-III prostate cancer	⋅ Contextual factors that influence TDM ⋅ Driver and Facilitator factors ⋅ Conflicts between TDM factors
Weda 2023 the Netherlands [86] Testicular (n = 12)	Grounded theory Semi-structured interview	0% women Age: 28-49y; $\overline{X }=$ 33y T: 0-5y	To understand […] survivors’ transition […] to long-term survivorship	⋅ Living Beyond the Sword of Damocles ⋅ Getting on with one’s life

* *T* Treatment, *O* Operation, *C* chemotherapy, *D* Diagnosis

The number of themes emerging from studies with a specific focus was 114 in total: 56 themes from studies focused on experiences with treatment, services and self-management, 14 themes from those exploring late effects, 13 themes from the studies centred on the working situation, 11 themes from the studies on psychological distress, and 20 themes from those studies centred in other topics.

### Results of synthesis: main thematic analysis

**Fig. 2:**
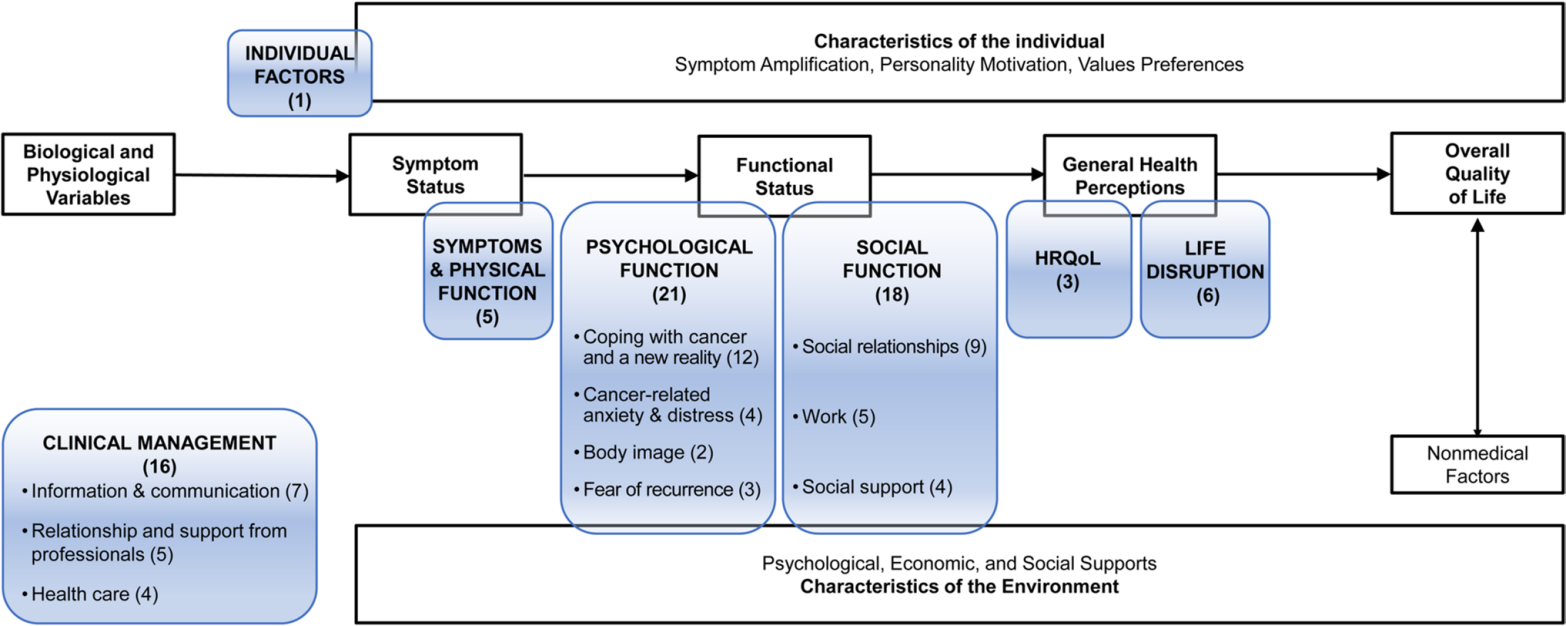
Overview of the results of the main thematic analysis of qualitative studies with a generic focus within the Wilson and Cleary HRQoL framework [42]. Clear boxes show the framework developed by Wilson and Cleary [42]. Coloured boxes show the categories and subcategories that emerged from the thematic analysis (number of themes/subthemes identified in the primary studies)

Figure 2 shows in clear boxes the framework developed by Wilson and Cleary [42]. It conceives HRQoL as a multidimensional construct encompassing five components (biological and physiological variables, symptom status, functional status, general health perceptions and overall quality of life), as well as the characteristics of the individual and the environment that affect these components. Coloured boxes show the categories and subcategories that emerged from the thematic analysis of themes and subthemes identified in the primary studies, and they are placed within the original components of the pre-existing framework. The category of Clinical Management falls outside of any component, three categories (Symptoms & Physical function, Psychological function and Social Function) are mainly covering the component of Functional status, and two categories (HRQoL and Life Disruption) are within the General Health Perceptions component.

Table 3 shows the evidence from the 16 studies with a generic focus aiming to explore outcomes, experiences, needs, concerns, worries, or quality of life impact relevant for people surviving cancer. It presents a mapping of all the themes and subthemes (descriptions or verbatims when subthemes were not reported) aggregated into categories. Some of the themes were categorized into more than one category or subcategory, according to the content of the subthemes or verbatims.

**Table 3:** Themes and subthemes (or verbatims) distributed into categories

**A. CLINICAL MANAGEMENT (16 themes)**	
***A.1. Information and communication (7 themes)***	
*Need for broader supportive care [50]	Need to know where to go and whom to turn to (Information about available care options and information about whom to turn to with questions and problems)
Needs regarding (medical) information and care [50]	Need for tailored patient information, available at one location (Information tailored to individual’s situations, information tailored to individual’s needs and information in understandable language); Need for periodic and additionally flexible follow-up (Periodic follow-up checks provide reassurance and additional flexible follow-up when needed)
Enough knowledge to understand what is happening [45]	Tailored information about treatment and consequences; Tailored information from specialists and peers about side effects and how to prevent them; HCPs to contact when in need for more information (reinformed)
Problematic events [52]	Being informed about the diagnosis and any challenges that arose due to it and explanation of the course cancer could take
To be met with interest and support [45]	To see, listen to and make sure information is tailored to their need; Hope and predict ability; To bring along support to information meeting
Overwhelmed by information [56]	“Therefore, you may have to consider how to distribute the information, there is an unlikely amount of information in the beginning (…) but it could be a good idea to reduce it and mete out in small measures when you need it”
Selecting information that enhances self-management strategies [54]	Limit the amount of prognostic information they received; Individual disease trajectory cannot be determined with any certainty; Increase their chances for a prolonged period of life, or to ease symptoms by using complementary and alternative therapies; Other long-term survivors searched for literature describing positive patient cases written by cancer survivors
***A.2. Relationship and support from professionals (5 themes)***	
Partnership with the multidisciplinary team [44]	Partnership between members of the team and the patient through the recovery process; Openness from the team supported individual adjustment at the psychological and practical level; Easy access to information from the team
Experiences with the professional care and the care trajectory [58]	‘No, I think that if a doctor tells me something, and I have the feeling that he is telling me the truth, then I don’t feel the need to be on the computer. I don’t need to search books to see if it’s true. … It only makes you feel uncertain.’
Talking about mental well-being [56]	“There is a massive absence of mental support. Socio-economically, it might have made a lot of sense to help people return after treatment. It can be some support groups or individual conversations after the treatment as you do with parents who lose a child”
*Bodily and mental loneliness [49]	Information and timing mismatch
After chemotherapy phase [46]	Unmet needs (Receiving a longer aftercare period. Receiving information about the total duration of side effect, Receiving emotional support)
***A.3. Health care (4 themes)***	
Healthcare factor [52]	Cancer type; Treatment type; Rapport with clinicians; Health literacy; Health resources
*Environmental factors [52]	Health provider usage
Treatment-related issues [59]	Posts referred to prostate cancer medications and treatment-related consequences
Treatment as a paradox [53]	Reflections on treatment; Treatment after-effects
**B. SYMPTOMS AND PHYSICAL FUNCTION (5 themes)**	
Physical functioning [59]	"Next week I’m taking up sport again.… Since my operation, I walk twice a day (for half an hour) [along with] my other activities.… Life continues, and it’s wonderful!"
*Bodily and mental loneliness [49]	Bodily and mental challenges
*Body and physical issues [55]	Major surgery for minor symptoms: perception that the therapeutic measures are disproportionate; A reduction in physical quality of life: The consequence of age or of cancer treatments?
*Protection for safety reasons [54]	Heavy symptom burden and a variety of late complications
*Negative manifestations of cancer survivorship [51]	Physical/bodily side effects;
**C. PSYCHOLOGICAL FUNCTION (21 themes)**	
***C.1. Coping with cancer and a new reality (12 themes)***	
Self beyond cancer [44]	Altered concept of self; Sense of resilience; Actions to regain roles and identity; Assumption of psychological approaches to living with cancer; Developing expert knowledge; Altruistic actions, empathize with other’s situations; Willingness to participate in research
Dealing with prostate cancer and treatment [58]	‘On the one hand I was ok with it, but on the other hand I was, like, “why me?” And then I think, “well, so be it. I can’t change it. It is what it is” ‘
Reviewing one’s perspectives on life-influenced coping strategies [57]	“I’ve had enough people to talk to and the intimacy with friends and family did me good”
*New centre of gravity in everyday life [49]	Reorientation of daily occupations
A plan to build/base the new life [45]	Someone to contact when in need; Use of humour, direct language; Accept the new situation, body changes; Use own experiences to help fellow stranger
Adversarial growth [53]	Re-establishing normality; Acceptance
Finding a new balance [50]	Coping with uncertainty; Changed perspective on life, re-evaluation of close relationships and changed personality; Towards no longer being a patient
Living Beyond Colorectal Cancer: Impact and Benefit [47]	Living with the impact of colorectal cancer; Striving to find benefits in the experience of cancer
Personal factors [52]	Personality; General self-efficacy; Responsibilities; Mentality; Resilience; Life events
Reclaiming one’s role [48]	“I don’t know, but at the beginning, I did, and now it has become normal, and I don’t pay attention to it anymore. I don’t think any more of those who say: ‘Look at that unfortunate guy’, so I don’t have any fears or problems”
Putting changes into action [51]	Prioritizing one’s self; Professional changes; Helping others
Changes in perceptions of self [51]	A better version of myself; A stronger sense of capability;
***C.2. Cancer-related anxiety & distress (4 themes)***	
Emotional fluctuations [53]	Challenges to identity; Long-term worries
Psychological and social role functioning [59]	Bloggers reported feeling surprised or shocked upon hearing of the possibility of having prostate cancer and when receiving a confirmatory diagnosis of prostate cancer
Feeling unpleasant emotions [48]	“The aftermath of the operation was hard. When I looked at myself in the mirror, I didn’t recognise myself; I didn’t know who I was”
Accepting a life with the “without” to survive [48]	“It was bad at frst. I was going a little crazy. I was already thinking of the worst. It happened… I still have a knife like that [he opens his hands to show the length of the knife]”
***C.3. Body image (2 themes)***	
Changes in the body [57]	Invisible body changes; Visible body changes
*Body and physical issues [55]	Impact on body image and on feminine identity
***C.4. Fear of recurrence (3 themes)***	
The Shadow of Colorectal Cancer: Fear and Vigilance [47]	Living in the shadow of colorectal cancer; Striving for vigilance
Dealing with a switch in prognosis [50]	Mixed feelings and emotions regarding prognosis switch; Facing an uncertain future
*Negative manifestations of cancer survivorship [51]	Fear of cancer recurrence
**D. SOCIAL FUNCTION (18 themes)**	
***D.1. Social relationships (9 themes)***	
*Bodily and mental loneliness [49]	Relationship and partnership (Sexual relations)
*Changed relationships with partners [57]	Sexual challenges
*Changes in social life [57]	The importance of social networks
*Changed relationships with partners [57]	Vulnerable relationship
Involvement of and with others [58]	Fellow patients; Personal relationships
*The impact of cancer experience on social life [55]	The evolution of social activities: The impact of age and OC treatments; Providing care to others: Social adjustments after OC experience
*Protection for safety reasons [54]	The effects of the patients profound symptom burden negatively influenced their social relationships; Patients and the caregivers explained that their family roles changed
Getting the hang of communication again [48]	“I was a chatterbox before the operation, but not so much now”
*Negative manifestations of cancer survivorship [51]	Negative impact on relationships
***D.2. Work (5 themes)***	
*New centre of gravity in everyday life [49]	The meaning of work
*Changes in social life [57]	The importance of work
*The impact of cancer experience on social life [55]	The impact of OC experience on participants professional careers
Searching for meaningful activities [54]	Impaired health due to the disease, often leading to a working disability that also caused psychological vulnerability; Faced various obstacles when trying to returning to work
*Putting changes into action [51]	Professional changes
***D.3. Social support (4 themes)***	
Enablers [44]	Societal attitudes to cancer; Willingness to demystify the stigma of cancer; Social support to achieve sense of normality; Personal goals and targets; Return to work
*Need for broader supportive care [50]	Need for psychosocial support (Practical and personal information, psychological information and support, access to peer support and work-related information and support); Need for support for close relatives (Support in dealing with consequences of disease)
*Environmental factors [52]	Social support; Community support; Travel
Reactions to experiences [59]	They [bloggers with prostate cancer] forewarned each other about future challenges. They used the blog to alert other men, to urge their Association’s president to take stronger political action, and to search for solutions
**E. HEALTH-RELATED QUALITY OF LIFE - HRQoL (3 themes)**	
Impacts on quality of life [59]	The data illustrated how prostate cancer affected men’s functioning
Impact of prostate cancer [58]	‘No, I guess I function quite well. I do the same things I did 10 years ago. I am still …, I feel healthy and vital, and I am still actively doing different kinds of things.’
The fine details to quality of life [56]	“The nuisance I have after cancer, I have learned to live with. You just drink something more or you have to chew the food something extra.” […] “When I do not have the joy of going to work, I have to take care of myself and get the best out of life. So, I can retire, it’s just a matter of how big the pension will be”
**F. LIFE DISRUPTION (6 themes)**	
Self-identification [59]	The bloggers usually identified themselves by their diagnosis, results, treatment method, and rehabilitation; only very few bloggers mentioned their social identities, whether as husbands, fathers, or professionals. Their ‘patient’ identity or health status was described primarily in terms of medical metrics […]
The impact of cancer experience on perception of life [55]	"Becoming mindful"; Understanding ovarian cancer experience from the patient trajectory perspective
Challenges to proceed with life as prior to metastatic cancer [50]	Demands and expectations to resume life again; Persistent complaints and new problems in different life domains High demands in several life domains; High expectations of oneself; Assumptions about being cured by surroundings; Persistent physical and psychological complaints; Late effects of treatment; Issues in returning to work; Negative influence on social life; Problems felt by close relatives
The Vestiges of Colorectal Cancer: Loss and Control [47]	Living with loss; Striving to regain, maintain and reconceptualise control
Transitions—Cured but not healed [56]	“In the past, I was a food, wine, and beer connoisseur. I am not anymore. If I smell and taste wine, then the nuances are gone. […] it has had the advantage that where wine may well cost 150–200 kroner, now I can settle for one to 50, you have to see the positive in it (laughs)”
Changes in perceptions of life [51]	The future is uncertain and you only live once; Greater appreciation of life
**G. INDIVIDUAL FACTORS (1 theme)**	
Pre-existing factors [52]	Age; Gender; Chronic conditions; Employment; Finances; Deprivation; Relationship status; Urban life

^*^Themes categorized into more than one category or subcategory according to the content of the subthemes

*CC* Colorectal cancer, *HCP* Health-care professionals, *OC* Ovarian cancer

#### A. Clinical management

A1. Information and communication: The seven themes included in this subcategory highlight the need for clear, quality, tailored, and timely information and communication with health professionals. For example, people surviving melanoma expressed the preference for receiving information that specifically applies to their (medical) situation instead of general information, and for more relevant information from their own perspective:

“I’d like the information to be provided from my—the patients’—perspective. Sometimes it can be too clinical from the doctors’ perspective.” [50]

A2. Relationship and support from professionals: Five themes consider the relationship with health professionals, their emotional and practical support, and the expectations on their actions to connect with other health-care professionals. A study of people surviving colorectal cancer pointed out that the openness offered by a multidisciplinary team supported their adjustment at the psychological and practical level:

“I wanted to know when I could try to go sailing again, I thought that it would be a daft question to ask the Dr. X sailing is so important to me, but I was wrong, she was happy to give me the advice that I needed” [44]

A3. Health care: The 4 themes included in this subcategory refer to health care resources and therapies. The debilitating symptoms experienced by women surviving cervical cancer [53] challenged the idea that treatment is a cure, and it was viewed as both a cure and an illness.

#### B. Symptoms and physical function

This physical category included 5 themes related to symptoms burden, functional effects and bodily challenges. People surviving brain cancer described a heavy symptom burden, including fatigue and reduced cognitive capacity:

“The treatment has caused several late complications and significant nerve damage, to such an extent that I’m considering discontinuing my treatment plan, and then following the strategy ‘wait and see’ … I have every late complication you can imagine.” [54]

#### C. Psychological function

C1. Coping with cancer and the new reality: Themes with coping content were the most frequent (12 of 21): acceptance of the new situation, re-establishing normality, finding a new balance, finding benefits in the experience of cancer, and resilience towards the situation. A woman surviving colorectal cancer expressed:

“I don’t know how it affects other people, […] I’m counting my blessings; it’s not stopping me getting around.” [47]

C2. Cancer-related anxiety and distress: Includes 4 themes mainly expressing uncertainty and long-term worries. In a study of women surviving cervical cancer, the transition from being a patient to a survivor was described as a time that challenged one’s identity and purpose, with feelings of isolation after being discharged [53].

C3. Body image: The two themes included in this sub-category were identified from two studies in different tumour locations. Women surviving ovarian cancer expressed a great deal of dissatisfaction with their body image and development of psychological problems due to scars from the surgery:

“I say to myself “Well even at fifty-nine years old, I have the right to be a woman again and to feel like a woman and there you have it.”" [55]

C4. Fear of recurrence: Few themes emerged from two studies, one in people surviving melanoma dealing with a switch of prognosis, and another on colorectal cancer. In the latter study, a participant expressed:

“You’ll never have a headache again, it’ll be a brain tumour […] you’ll think worst case scenario, and that is me, I have turned into that person … you wake in the morning, how am I? … Am I okay? …” [47]

#### D. Social function

D1. Social relationships: The nine themes included in this subcategory highlighted the impact on the family, social network, and social activities. For example, a woman surviving breast cancer commented on how their social life changed:

‘I notice that I don’t want much contact with the people who don’t ‘give me’ anything. Because I think, my life is too short to have relations with people who only suck energy out of me’ [49].

D2. Work: There were 5 themes centred on how work is a relevant part of relationships. As long as women surviving breast cancer managed to meet the expectations during a workday, they all described employment as a meaningful activity, that seemed to give them energy and could represent a ‘free space’ [49].

D3. Social support: 4 themes described how a sense of normality is commonly obtained through a range of sources such as family, friends and work colleagues. People surviving colorectal cancer mentioned family as support players [57]: women felt it was easier to talk about feelings with their friends and family while men thought these feelings were to be shared only with their partners.

#### E. Health-related quality of life (HRQoL)

Only 3 themes were categorized into HRQoL. Male informants surviving head and neck cancer described that if challenged in terms of health, one must compensate and recreate the quality of life:

“There has been a shift towards something more positive, she has joined my ritual of sea bathing, so there is some closeness around us that is new” [56].

#### F. Life disruption

This category included 6 themes highlighting self-identification by their oncological condition, assumptions from their surroundings about being cured, reconceptualizing control, or cured but not healed. For example, the adoption of a new perspective of life post-ovarian cancer had a positive impact for some women, while for others it remains too difficult to implement:

“I still think it must inevitably change people’s perception of life so, maybe there are those who, on the contrary, were initially negative: “My God, I was sick.” Then there are others. For me it’s: “My God, I’m alive! So, there you have it!”" [55]

#### G. Individual factors

Pre-existing individual factors, including age, gender, chronic conditions, employment, finances, relationship status and urban/rural setting were only identified in one study [52], where participants in a better financial position wished they had been better informed on private treatment options to expedite management.

### Results of synthesis: secondary thematic analysis of studies with specific objectives

The results of the thematic analysis from studies with specific objectives are shown in supplementary tables. From the 12 qualitative studies focused on treatment and self-management (Supplementary Table 4), most of the 56 themes identified fit in the same categories from the above-mentioned thematic analysis, except for a new category entitled ‘Healthy Lifestyle’. The 13 themes from studies exploring late effects (Supplementary Table 5) mainly highlighted psychological function, while the 13 themes identified in studies centred on the work situation (Supplementary Table 6) pointed out the importance of social function, with very few themes in the physical function category. Finally, the studies focused on psychological distress (Supplementary Table 7) identified a total of 11 themes distributed among the categories of the main thematic analysis.

## Discussion

The review of the existing evidence of qualitative research focused on outcomes, needs, experiences, preferences, concerns and quality of life of people in Europe who survived cancer identified 43 studies fulfilling inclusion criteria from the 18,256 articles found in the search. Main thematic analysis of more than 50 themes and subthemes extracted from the 16 qualitative studies with a generic focus showed that most fitted within Wilson and Cleary’s domain of Functional status (‘Symptoms and Physical Function’, ‘Psychological Function’ and ‘Social Function’ categories), with clearly fewer themes fitted in the domain of General Health Perceptions (‘HRQoL’ and ‘Life Disruption’ categories). On the other hand, the ‘Clinical Management’ category emerged as a prominent concern beyond the domains of this framework. Furthermore, among the 27 qualitative studies with specific objectives, the construct most frequently explored was the experience with treatment, services and self-management, which is the focus of 12 studies, raising 56 themes.

The thematic analysis showed the relevant impact of cancer in ‘Psychological Function’ in studies with both generic and construct-specific objectives. The themes related to coping with cancer and the new reality were the most frequent among the studies with a generic focus, but, in those with a specific focus, the themes about cancer-related anxiety and distress and fear of recurrence were mentioned more frequently than positive ones. Our results are consistent with a systematic review of the trajectories of clinically relevant distress in adults with cancer [28], which found symptom burden as the most consistent predictor of persistent distress and highlighted the relevance of multi-disciplinary mental health interventions. Another systematic review on screening for psychosocial well‐being and care needs [27] identified some studies showing benefits, but the metanalysis did not demonstrate efficacy. Lastly, fear of recurrence was explored in other systematic reviews showing that its trajectory was predicted by psychological characteristics [34], and the importance of its management as a diverse emotional experience described in trauma-like terms by some individuals [33].

The ‘Social Function’ impact of cancer for survivors is clearly shown in the studies with a generic focus by mentions of bodily and mental loneliness, vulnerable relationships, change of family roles, the need of adjustments in social life and social support to achieve a sense of normality. Work emerges as a particularly relevant aspect, including the impact on working disability, the limitation of professional careers and working relationships. On one hand, the publication of three qualitative studies centred on the working situation [75–77] also supports the importance of this aspect. On the other hand, the themes emerging from these studies identified difficulties and worries (symptoms burden, attitude of colleagues, and lifestyle modifications) [76], benefits (mood improvement [76], engagement and socialisation [77]), sources of motivation for continuation of work life [76], and the need for adjustments of work tasks [75]. All these results support that return to work strategies need to be included in cancer survivorship programs. Along these lines, a systematic review on predictive factors for return to work in European people surviving cancer found that risk factors can be identified earlier in the patient pathway, and programs should focus on early detection [21].

The few themes included in the category of ‘Symptoms & Physical Function’ suggest that this area of high relevance in the traditional framework of HRQoL [42] has less prominence for people surviving cancer, despite the persistence of symptoms and physical function limitations in this population. A systematic review on cancer-related fatigue [25] found high prevalence of this symptom, which presents milder severity among the working, compared to non-working, survivors. This difference could generate a significant health disparity, highlighting the necessity of specific policies to support the return to normalcy.

Within the Wilson and Cleary domain of General Health Perceptions, themes emerged more frequently in our category of ‘Life Disruption’ than in ‘HRQoL’. The connection between both categories is consistent with findings from a systematic review on the experience of HRQoL in people surviving cancer, reflecting how a new sense of normality is motivated by the privilege of being alive [87]. On the other hand, themes identified in our review within the category of ‘Life Disruption’ also refer to negative aspects, such as self-identification by their oncological condition [59] or challenges to resume their life [50].

The main thematic analysis illustrates that, in the ‘Clinical Management’ category, the need for tailored information and communication with health professionals is the most clearly and consistently mentioned, followed by relationship with them, their emotional and practical support, and health care resources. The publication of numerous qualitative studies with purposes centred on the experiences with treatment, services, and self-management [60–71], also confirm the relevance of these aspects from the perspective of stakeholders other than patients, such as researchers, clinicians or health managers. Our results are consistent with a mixed-method systematic review of unmet care and support needs among Japanese people surviving cancer [16], in which individuals reported insufficient tailor-made information, and care and support from professionals. Another systematic review on unmet needs [18] found that these are higher in countries with less robust health systems, and in people with less time since diagnosis. Subsequently, even though ‘Clinical Management’ was not included as a component of the Wilson and Cleary framework, and it is generally understood as part of the patient’s experience instead of a health outcome, our synthesis of qualitative studies suggests that it is a relevant aspect from the patient’s perspective in the patient-centred care approach. Furthermore, management of therapeutic regimens and self-management was emphasized as a gap of the Wilson and Cleary framework [43].

Results presented in this review should be interpreted carefully. First, publication bias could affect studies reporting findings on traditional domains (e.g. pain, fatigue, anxiety) because they may be considered of no interest, and these could be underrepresented in our search restricted to peer-reviewed articles. Second, many of the included studies had a specific objective, not aiming to widely identify quality of life-related issues relevant to people surviving cancer. However, to avoid overrepresentation of results in the synthesis from these studies, the main thematic analysis was centred on results from studies with a generic focus. Finally, the studies that fulfilled the inclusion criteria do not represent all the EU-27 countries, nor all the associated ones. There is published evidence from only 7 countries of the EU-27, 3 of the associated countries (Sweden, Turkey, Israel) and the UK. People from more than half of the EU-27 countries are, therefore, not represented in the published evidence collected in this systematic review. The lack of information coming from people surviving cancer of southern and eastern European regions is remarkable.

Lastly, as most of the studies with a generic focus are from the last 5 years (11 out of 16), a strength of this systematic review is that the results capture the current situation of people surviving cancer: new therapies, new timelines, or new management procedures in specific units. It is worth mentioning that the majority of the studies (40 out of 43) could be considered of good quality, and 6 fulfilled positively the whole SURE checklist. The information least reported in the studies was the ‘relationship between the researcher and the participant’, even though it is also part of previous checklists for qualitative studies [88, 89]. Considering how recent the included studies are and their good methodological quality, findings from this review could be valuable to select domains relevant to people currently surviving cancer in future PROMs, as well as they have been useful to establish recommendations for the ongoing development of the EUonQoL toolkit.

## Conclusion

In conclusion, results on this systematic review clearly showed that social and psychological function domains predominate over physical symptoms and function domains among people surviving cancer; and they also add the identification of specific needs in clinical management, such as information and communication, relationship with and support from health professionals, and health care. These aspects, usually not present in the existing HRQoL instruments for people surviving cancer, due to being understood as content of patient-reported experience measures, appear now as a potentially relevant domain in the patient-centred care approach. The findings from this review have helped in the EUonQoL toolkit development, which will be validated using data from a survey conducted in all European countries. It is necessary that PROMs reflect all the identified domains to cover all the aspects considered relevant by people currently surviving cancer to devise clinical, societal, and healthcare policymaking systems.

## Supplementary Information

Below is the link to the electronic supplementary material.

Supplementary file1 (DOCX 106 KB)

## Data Availability

The datasets generated and/or analysed during the current study are available from the corresponding author on reasonable request.
