# Supplementary material for: Systematic review of the needs and health-related quality of life domains relevant to people surviving cancer in Europe
Source: Qual Life Res. 2025 Jan 23;34(4):913–36. doi: 10.1007/s11136-024-03884-w (PMC11982114; doi:10.1007/s11136-024-03884-w)
Supplement: Supplementary file 1 — Supplementary file1 (DOCX 106 KB) [file 11136_2024_3884_MOESM1_ESM.docx]

**SUPPLEMENTARY INFORMATION**

**Systematic review of the needs and Health-Related Quality of Life domains relevant to people surviving cancer in Europe**

Clara Amat-Fernandez^1,2,3^, Olatz Garin^1,2,3^, Ricardo Luer^1^, Yolanda Pardo^1,2,4^, Renata Briseño^1^, Catalina Lizano-Barrantes^1,5^, Leslye Rojas-Concha^6^, Melissa Thong^7^, Giovanni Apolone^8^, Cinzia Brunelli^8^, Augusto Caraceni^9^, Norbert Couespel^10^, Nanne Bos^11^, Mogens Groenvold^12^, Stein Kaasa^13^, Gennaro Ciliberto^14^, Claudio Lombardo^15^, Ricardo Pietrobon^16^, Gabriella Pravettoni^17^, Aude Sirven^18^, Hugo Vachon^19^, Alexandra Gilbert^20,21^, Galina Velikova^20,21^, Montse Ferrer^1,2,3^, and the EUonQoL Working Group†.

1. Health Services Research Group, Hospital del Mar Research Institute, Barcelona, Spain.
2. CIBER en Epidemiología y Salud Pública, CIBERESP. Madrid, Spain.
3. Department of Medicine and Life Sciences. Universitat Pompeu Fabra. Barcelona, Spain.
4. Department of Psychiatry and Legal Medicine, Universitat Autònoma de Barcelona. Barcelona, Spain.
5. Department of Pharmaceutical Care and Clinical Pharmacy, Faculty of Pharmacy, Universidad de Costa Rica, San Jose, Costa Rica.
6. Palliative Care Research Unit, Department of Geriatrics and Palliative Medicine GP, and Frederiksberg Hospital, University of Copenhagen, Denmark.
7. Unit of Cancer Survivorship, Division of Clinical Epidemiology and Aging Research, German Cancer Research Center (DKFZ). Heidelberg, Germany.
8. Palliative Care, pain therapy and rehabilitation Unit, Fondazione IRCCS Istituto Nazionale Dei Tumori-Milano, Milan, Italy.
9. Università Degli Studi Di Milano, Milan, Italy.
10. European Cancer Organisation (ECO), Brussels, Belgium.
11. Netherlands Institute for Health Services Research (Nivel), Utrecht, The Netherlands.
12. Department of Public Health, University of Copenhagen, Copenhagen, Denmark.
13. Oslo Universitetssykehus HF, Oslo, Norway.
14. IRCCS National Cancer Institute “Regina Elena” Rome, Italy (on behalf of Digital institute for cancer outcomes research (DIGICORE), Brussels, Belgium).
15. Organisation of European Cancer Institutes, Brussels, Belgium.
16. SporeData OÜ, Tallinn, Estonia.
17. Istituto Europeo Di Oncologia IRCCS, Milan, Italy.
18. Unicancer, Paris, France.
19. European Organisation for Research and Treatment of Cancer, Brussels, Belgium.
20. Leeds Institute of Medical Research at St James’s, University of Leeds, Leeds. United Kingdom.
21. Leeds Teaching Hospitals NHS Trust, Leeds. United Kingdom.

†Members of the EUonQoL Working Group: Massimo Costantini, Madeline Pe, Galina Velikova, Chiara Marzorati, Antonio Tanzilli, Morten Aagaard Petersen, Olatz Garin, Aline Machiavelli, Joachim Widder, Helidon Nina, Philip Debruyne, Ivaylo Petrov, Vesna Ramljak, Maria Krini, Tomas Kazda, Helle Pappot, Liina Pääbo, Vahur Valvere, Johanna Mattson, Ann Bredart, Carole Boulec, Mariaalice Borinelli-Franzoi, Ekaterina Kldiashvili, Christian Brandts, Nicole Erickson, Volker Arndt, Olga Balaoura, Horvath Orsolya, Claire Donohoe, Alessandro Rizzo, Andrea Pace, Sandra Lejniece, Audrius Dulskas, Vadim Pogonet, Lonneke van de Poll, Marianne Grønlie Guren, Iwona Ługowska, Maria Litwiniuk, Maria José Bento, Tudor Ciuleanu, Milana Mitrić, Ivica Ratosa, Michal Chovanec, Maria Vieito, Héctor Aguilar, Eva Ruiz, Karin Ahlberg, Eda Tanrikulu Simsek, Mahmut Gumus, Inke Minnée-van Braak, Caitriona Higgins, Laura Pinnavaia, Carina Dantas, Tapani Kalmi, Leslye Rojas-Concha.

**Corresponding authors:**

Montse Ferrer (mferrer@researchmar.net) & Olatz Garin (ogarin@researchmar.net)

Health Service Research Group. Hospital del Mar Research Institute.

Barcelona Biomedical Research Park, office 144.

88 Doctor Aiguader street, 08003 Barcelona, Spain.

Telephone +34 933 160 763.

**Supplementary table 1.** List of European and associated countries in the EUonQoL project

| 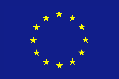  **European and associated countries** | | |
| --- | --- | --- |
| Albania | Germany | North-Macedonia |
| Armenia | Greece | Norway |
| Austria | Hungary | Poland |
| Belgium | Iceland | Portugal |
| Bosnia and Herzegovina | Ireland | Romania |
| Bulgaria | Israel | Serbia |
| Croatia | Italy | Slovenia |
| Cyprus | Kosovo | Slovakia |
| Czechia | Latvia | Spain |
| Denmark | Lithuania | Sweden |
| Estonia | Luxembourg | Tunisia |
| Faroe Islands | Malta | Turkey |
| Finland | Moldavia | Ukraine |
| France | Montenegro | United Kingdom |
| Georgia | Netherlands |  |

**Supplementary table 2.** Search Strategy used for the literature review in PubMed and Scopus.

| **PubMed** |
| --- |
| ("Patient*"[Text Word] OR "Survivor*"[Text Word] OR "Palliative Care"[Text Word] OR "Patient*"[Mesh] OR "Survivor*"[Mesh] OR "Palliative Care"[Mesh]) |
| AND ("Neoplasms"[Mesh] OR "post-cancer" [Title/Abstract] OR "postcancer" [Title/Abstract]) |
| AND ("Quality of Life"[Mesh] OR "perceived health"[Text Word] OR "health status"[Text Word] OR "well-being" [Text Word] OR "wellbeing"[Text Word] OR "Patient Reported Outcome Measures"[Mesh] OR “health-related quality of life”[Text Word] OR “health related quality of life”[Text Word] OR “patient-reported outcome*” [Text Word] OR “patient reported outcome*” [Text Word]) |
| AND (“relevan*”[Text Word] OR “import*”[Text Word] OR “preferences”[Text Word] OR “feelings”[Text Word] OR “needs”[Text Word] OR “issues”[Text Word] OR “concerns”[Text Word] OR “worries”[Text Word] OR “difficulties”[Text Word] OR “limitations”[Text Word] OR “experiences”[Text Word] OR “problems”[Text Word]) |
| FILTERS: English; Publication Date since 2013 |

| **Scopus** |
| --- |
| (TITLE-ABS-KEY ( "Patient*" OR "Survivor*" OR "Palliative Care" ) ) |
| AND ( TITLE-ABS-KEY ( "Neoplasms" OR "post-cancer" OR "postcancer" ) ) |
| AND ( TITLE-ABS-KEY ( "Quality of Life" OR "perceived health" OR "health status" OR "well-being" OR "wellbeing" OR "Patient Reported Outcome Measures" OR "health-related quality of life" OR "patient reported outcome*" OR "health related quality of life" OR "patient-reported outcome" ) ) |
| AND ( TITLE-ABS-KEY ( "relevan*" OR "import*" OR "preferences" OR "feelings" OR "needs" OR "issues" OR "concerns" OR "worries" OR "difficulties" OR "limitations" OR "experiences" OR "problems" ) ) |
| AND PUBYEAR > 2012 |
| AND ( EXCLUDE ( AFFILCOUNTRY , "United States" ) OR EXCLUDE ( AFFILCOUNTRY , "China" ) OR EXCLUDE ( AFFILCOUNTRY , "Canada" ) OR EXCLUDE ( AFFILCOUNTRY , "Australia" ) OR EXCLUDE ( AFFILCOUNTRY , "Japan" ) OR EXCLUDE ( AFFILCOUNTRY , "South Korea" ) OR EXCLUDE ( AFFILCOUNTRY , "Switzerland" ) OR EXCLUDE ( AFFILCOUNTRY , "Brazil" ) OR EXCLUDE ( AFFILCOUNTRY , "India" ) OR EXCLUDE ( AFFILCOUNTRY , "Taiwan" ) OR EXCLUDE ( AFFILCOUNTRY , "Iran" ) OR EXCLUDE ( AFFILCOUNTRY , "Hong Kong" ) OR EXCLUDE ( AFFILCOUNTRY , "Singapore" ) OR EXCLUDE ( AFFILCOUNTRY , "Russian Federation" ) OR EXCLUDE ( AFFILCOUNTRY , "Mexico" ) OR EXCLUDE ( AFFILCOUNTRY , "New Zealand" ) OR EXCLUDE ( AFFILCOUNTRY , "Malaysia" ) OR EXCLUDE ( AFFILCOUNTRY , "Saudi Arabia" ) OR EXCLUDE ( AFFILCOUNTRY , "Egypt" ) OR EXCLUDE ( AFFILCOUNTRY , "South Africa" ) OR EXCLUDE ( AFFILCOUNTRY , "Thailand" ) OR EXCLUDE ( AFFILCOUNTRY , "Chile" ) OR EXCLUDE ( AFFILCOUNTRY , "Indonesia" ) OR EXCLUDE ( AFFILCOUNTRY , "Colombia" ) OR EXCLUDE ( AFFILCOUNTRY , "Argentina" ) OR EXCLUDE ( AFFILCOUNTRY , "Pakistan" ) OR EXCLUDE ( AFFILCOUNTRY , "Jordan" ) OR EXCLUDE ( AFFILCOUNTRY , "Lebanon" ) OR EXCLUDE ( AFFILCOUNTRY , "Nigeria" ) OR EXCLUDE ( AFFILCOUNTRY , "United Arab Emirates" ) ) |

**Supplementary table 3.** Quality appraisal of the included qualitative studies.

|  | Does the study address a clearly focused question/hypothesis | Is the choice of qualitative methodology appropriate | Is the sampling strategy clearly described and justified | Is the method of data collection well described | Is the relationship between the researcher(s) and participants explored | Are ethical issues explicitly discussed? | Is the data analysis/interpretation process described and justified? | Are the findings credible? | Is any sponsorship/conflict of interest reported? | Did the authors identify any limitations? |
| --- | --- | --- | --- | --- | --- | --- | --- | --- | --- | --- |
| Author, Year | D1 | D2 | D3 | D4 | D5 | D6 | D7 | D8 | D9 | D10 |
| Anderson (2013) [60] | 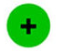 | 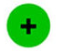 | 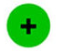 | 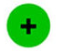 | 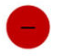 | 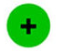 | 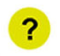 | 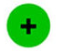 | 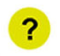 | 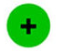 |
| Appleton (2013) [44] | 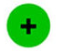 | 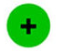 | 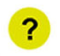 | 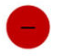 | 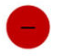 | 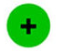 | 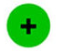 | 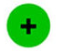 | 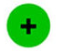 | 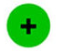 |
| Appleton (2014) [81] | 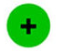 | 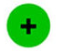 | 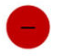 | 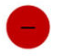 | 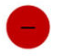 | 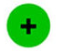 | 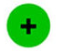 | 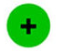 | 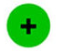 | 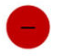 |
| Aunan (2021) [45] | 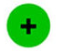 | 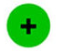 | 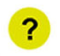 | 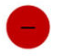 | 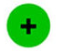 | 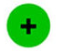 | 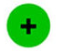 | 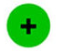 | 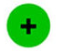 | 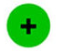 |
| Burden (2016) [61] | 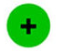 | 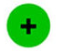 | 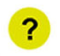 | 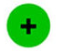 | 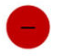 | 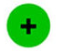 | 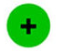 | 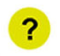 | 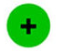 | 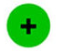 |
| Deery (2023) [82] | 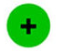 | 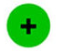 | 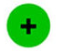 | 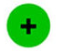 | 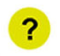 | 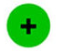 | 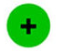 | 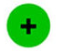 | 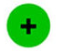 | 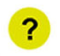 |
| den Bakker (2018) [46] | 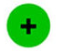 | 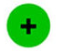 | 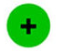 | 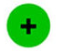 | 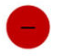 | 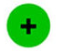 | 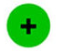 | 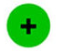 | 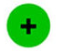 | 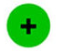 |
| Drury (2022) [47] | 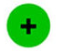 | 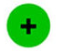 | 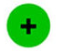 | 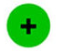 | 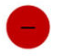 | 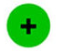 | 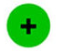 | 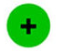 | 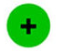 | 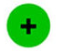 |
| Dunne (2018) [62] | 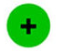 | 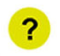 | 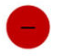 | 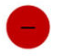 | 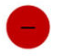 | 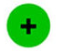 | 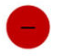 | 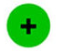 | 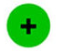 | 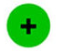 |
| Ghirotto (2023) [48] | 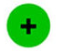 | 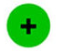 | 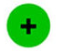 | 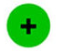 | 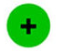 | 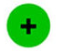 | 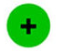 | 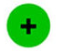 | 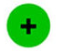 |  |
| Harrow (2014) [63] |  |  |  |  |  |  |  |  |  |  |
| Jakobsen (2018) [49] |  |  |  |  |  |  |  |  |  |  |
| Kamminga (2022) [50] |  |  |  |  |  |  |  |  |  |  |
| Koutoukidis (2017) [64] |  |  |  |  |  |  |  |  |  |  |
| Lagerdahl (2014) [83] |  |  |  |  |  |  |  |  |  |  |
| Levin-Dagan (2024) [51] |  |  |  |  |  |  |  |  |  |  |
| Liaset (2018) [75] |  |  |  |  |  |  |  |  |  |  |
| Mahmood (2024) [52] |  |  |  |  |  |  |  |  |  |  |
| Marshall-McKenna (2023) [65] |  |  |  |  |  |  |  |  |  |  |
| Matheson (2020) [78] |  |  |  |  |  |  |  |  |  |  |
| Millet (2022) [53] |  |  |  |  |  |  |  |  |  |  |
| Pallin (2023) [66] |  |  |  |  |  |  |  |  |  |  |
| Piil (2022) [54] |  |  |  |  |  |  |  |  |  |  |
| Puppo (2020) [55] |  |  |  |  |  |  |  |  |  |  |
| Regnier-Denois (2017) [67] |  |  |  |  |  |  |  |  |  |  |
| Reynolds-Cowie (2021) [79] |  |  |  |  |  |  |  |  |  |  |
| Samsøe (2022) [56] |  |  |  |  |  |  |  |  |  |  |
| Seibel (2023) [68] |  |  |  |  |  |  |  |  |  |  |
| Şengün İnan (2019) [84] |  |  |  |  |  |  |  |  |  |  |
| Şengün İnan (2020) [76] |  |  |  |  |  |  |  |  |  |  |
| Şengün İnan (2023) [80] |  |  |  |  |  |  |  |  |  |  |
| Stamataki (2015) [69] |  |  |  |  |  |  |  |  |  |  |
| Stuhlfauth (2018) [57] |  |  |  |  |  |  |  |  |  |  |
| Torp (2020) [77] |  |  |  |  |  |  |  |  |  |  |
| Treanor (2016) [72] |  |  |  |  |  |  |  |  |  |  |
| Trusson (2016) [73] |  |  |  |  |  |  |  |  |  |  |
| van Ee (2018) [58] |  |  |  |  |  |  |  |  |  |  |
| Voigt (2024) [70] |  |  |  |  |  |  |  |  |  |  |
| Wagland (2019) [85] |  |  |  |  |  |  |  |  |  |  |
| Weda (2023) [86] |  |  |  |  |  |  |  |  |  |  |
| Wennick (2017) [74] |  |  |  |  |  |  |  |  |  |  |
| Wollersheim (2021) [71] |  |  |  |  |  |  |  |  |  |  |
| Zanchetta (2016) [59] |  |  |  |  |  |  |  |  |  |  |

**Supplementary table 4.** Results of thematic analysis of studies focused on treatment, services and self-management.

| **A. CLINICAL MANAGEMENT (21 themes)** | |
| --- | --- |
| **A.1. INFORMATION AND COMMUNICATION (5 themes)** | |
| Expectations of health professionals’ actions [65] | Communication; Share information with other treating health-care professionals |
| Health care system and information needs [69] | Clarity of information; Quality of information; Information at the right time; Time spent with health care professionals |
| Health system and information [71] | Information about test results; Information about impotence treatment; Information about follow-up appointments; Information about additional prostate cancer treatment; Information about the initial treatment for prostate cancer |
| Information provisions [70] | Need for reliable, centered and tailored information; Need for positive stories of other patients |
| *Preferred components of self-management support [66] | Information and instruction about cancer and treatment side effects |
| **A.2. RELATIONSHIP AND SUPPORT FROM PROFESSIONALS (8 themes)** | |
| Support/information since the end of treatment[65] | Psychological/emotional |
| Lack of Awareness of Supportive Care Services [67] | No Individual Nurse Consultation; Incomplete memorization of list of support services; Usefulness of service not identified |
| Unmet Needs in Supportive Care Services [67] | Body image; Managing weight gain; Managing early menopause and side-effects; Psychological issues relating to post-treatment phases; Job-related issues; Fatigue/pain relating to treatment |
| Perceptions of and need for support [63] | Keeping it to themselves - everyone's different; no one's ever asked if I am still taking it; appropriate expertise |
| Barriers Stemming from Patients Mental Images of Supportive Care Services [67] | Mental associations with medical treatment/institution; Negative assumptions and need for personalisation/contact; Unpleasant experience with specific support department |
| Ideal support [65] | Practical/psychological/self-management |
| Support and monitoring are needed immediately after the initial treatment phase ends [66] | Participants highlighted the need for support immediately after treatment ends as this is when physical side effects are the most debilitating. |
| *Preferred components of self-management support [66] | Regular monitoring and support to help with adherence.; Advice and practical support around diet; Psychological strategies and information about managing psychosocial consequences.; Mode of self-management support |
| **A.3. HEALTH CARE (8 themes)** | |
| Limited Access to Services and Resources [67] | Geographical (mobility-distance); Lack of time; Too difficult to call for an appointment; Economic issues; Lack of problem anticipation |
| Ideal health services [65] | Hospital/primary care/other |
| Experiences of taking adjuvant endocrine therapy [63] | Remembering not to forget; it's a religion; living with the side effects |
| Comfort with technology [65] | Barriers reported |
| Concerns due to COVID [65] | Practical treatment concerns |
| The healthcare system [70] | Importance of a dedicated contact person; Need for understanding complications, HCPs, and quicker referral options |
| Quality of life questionnaires [70] | Need for personal feedback through questionnaires, with aid options |
| Remaining platform issue [70] | Need to take cultural differences, educational levels, security, and regular updates into account |
| **B. SYMPTOMS & PHYSICAL FUNCTION (8 themes)** | |
| Functional effects [69] | “I had a bit of a swelling on my leg, I went back and they treated it, it’s fine [clinician said], it will go down eventually…And I thought ‘Alright then, ok then’, and you kind of go home but at the same time I was probably wanting to sit down with somebody and say ‘right this is what you’ve been through, this is what you should do now and this is what it means.” |
| Taking control of symptom management [61] | Chemotherapy; stoma management |
| Symptom-related barriers [62] | Physical side effects and symptoms arising from treatment and its consequences; cognitive symptoms arising from treatment |
| Physical and daily living [71] | “I have to take into account that I go to the bathroom before I do any heavy lifting.” |
| Weight gain [61] | Weight gain as a consequence of surgical intervention or adjuvant chemotherapy was reported by four respondents. Fatigue, decreased mobility and alteration in dietary intake to manage stoma output were factors reported as contributing to weight gain. |
| Appetite swings [61] | “Well, over a period of time, it’s weird really because, 1 week I could be fine and next week I’d be way down again, and if I smelt food or see anybody eating, it used to put me completely off. And I could go days without eating” |
| *Health concerns/needs relating to age in survivorship [65] | Physical/symptom |
| Sexuality [71] | “I just have a problem with erections.” […] “I do miss it a lot.” |
| **C. PSYCHOLOGICAL (13 themes)** | |
| **C.1. COPING WITH CANCER AND A NEW REALITY (2 themes)** | |
| Cancer and life [70] | Need for help accepting and coping with physical and mental consequences; Need for help reintegrating in daily life and adapting to a new lifestyle |
| *The chronic nature of Low-grade non-Hodgkin’s lymphoma shapes perceptions of self-management [66] | Navigation, self-monitoring and proactive problem solving.; Maintaining a positive outlook; |
| **C.2. CANCER-RELATED ANXIETY & DISTRESS (5 themes)** | |
| Psychological [71] | "Yes of course, but I had a lot of nerves today. The whole week actually" |
| *Health concerns/needs relating to age in survivorship [65] | Psychological/emotional |
| Self-evaluation barriers [62] | Diminished self‐confidence; Interpersonal self‐evaluative concerns |
| *Emotional effects [69] | Uncertainty |
| *Emotional barriers [62] | Worries about posttreatment consequences; Low mood |
| **C.3. BODY IMAGE (2 themes)** | |
| *Emotional effects [69] | Altered body image; Fear of the sun |
| Emotions on changing physicality [61] | Preoperative changes; Post-operative changes |
| **C.4. FEAR OF RECURRENCE (4 themes)** | |
| *Emotional barriers [62] | Fear of recurrence |
| Meaning of follow-up care [68] | Impact of follow-up care on everyday life; Fear of and around scan; Sense of security and control; No impact on everyday life |
| Reasons for taking adjuvant endocrine therapy [63] | Lifeline to being cancer-free; doctor knows best |
| CEA-value [70] | Need for more focus on the psychological aspect; Need for interpreting assistance for the CEA-value; Need for anxiety management regarding CEA-value |
| **D. SOCIAL (7 themes)** | |
| **D.1. SOCIAL RELATIONSHIPS (2 themes)** | |
| Ongoing impact of curatively treated lung cancer in the family system: long-term and late effects [68] | Perceived long-term and late effects (physical, mental, social); No impact of long-term and late effects felt |
| *Effects on relationships [69] | Family relationships |
| **D.2. SOCIAL SUPPORT AND STIGMA (4 themes)** | |
| Structural barriers [62] | Financial resources; Access to appropriate health services |
| Family support needs [65] | Family-related concerns |
| Social networks enable self-management [66] | Healthcare professionals; Family support; Community cancer supports and peer support groups |
| Psychosocial needs during follow-up care [68] | […] cancer survivors also did not expect physicians to address issues related to QoL and psychosocial needs. [...] Psychosocial well-being was also viewed by some as primarily a self-care task. At times, psychosocial interventions were even negatively labelled with stigmas such as “being useless” (Survivor 19) or “dragging down” (Survivor 21). |
| **D.3. WORK (1 theme)** | |
| *Effects on relationships [69] | Working relationships |
| **E. LIFE DISRUPTION (1 theme)** | |
| Priorities in life post-treatment [65] | Finding a “new normal” |
| **F. HEALTHY LIFESTYLE (12 themes)** | |
| Medicalization of food [61] | Acceptance of oral nutritional supplements (ONS); Intolerance to ONS; Concerns about over reliance on ONS |
| Defining a healthy lifestyle [64] | Healthy eating and physical activity; mental, sexual, and psychological well-being |
| Factors influencing diet and physical activity [64] | "cognitive; physiological; emotional; social; and practical" |
| Needing to search for information [64] | "Desired advice, timing, and methods of delivery; Participants were interested in receiving reliable information about healthy lifestyle from their health care professionals or being directed to appropriate services by them" |
| Drivers for action [61] | Predominant drivers for dietary manipulation were: symptom management, a desire to maximise health and to manipulate weight to regain lost weight or to achieve a healthier perceived weight. |
| *The chronic nature of Low-grade non-Hodgkin’s lymphoma shapes perceptions of self-management [66] | Healthy diet and physical activity |
| Perceived need for advice on diet, physical activity and lifestyle UK [60] | “Yeah, the only verbal advice I was given was just to eat little and often, but I found myself eating a lot and often, my appetite actually improved…after the operation, I put a little bit of weight on.” |
| Beliefs about the role of diet, activity and lifestyle for reducing disease risk in the longer term [60] | Many participants were generally uncertain about the role of diet, activity and lifestyle in reducing the risk of the disease in the future. |
| Casual beliefs [60] | “The ironic thing was I was always very, very careful about my diet and I always exercised, I never really ate a lot of meat, vegetables, fruit, exercised like, you know, regularly, but I still got it” |
| Health maintenance actions [60] | “Some dietary advice would be extremely relevant I would have thought… I basically want to stack the odds in my favour…” |
| Patients interest in guidance on diet, activity and lifestyle to reduce disease risk and progression [60] | “I think we’re really all trying to get back to where we were… That for me was the challenge […] to get back to where I was before this all started…” |
| What are the preferred formats, timings and routes of delivery for guidance on diet, activity and lifestyle? [60] | “Personally what I would like to see at the end of treatment, some sort of survivorship audit where they’re looking at your diet, your lifestyle, how much exercise you’re getting, …and also the other aspects which doesn’t really get enough recognition is stress and anxiety is as much a side effect of your treatment as diarrhoea and neuropathy. I would like to see a package of those things at the end” |

*Themes distributed in two or more categories according to the content of the subthemes and verbatims.

**Supplementary table 5.** Results of thematic analysis of studies focused on late effects.

| **A. CLINICAL MANAGEMENT (1 theme)** | |
| --- | --- |
| **A.1. HEALTH CARE (1 theme)** | |
| Management [72] | Late effects experience acted as a prompt to seek health-care contact; experiences respect to referral and access to specialist services |
| **B. SYMPTOMS & PHYSICAL FUNCTION (1 theme)** | |
| *Onset and nature [72] | Cognitive impairment; Graft versus Host Disease; Urinary incontinence; Aches and pains; Fertility loss; Lymphedema; Menopausal symptoms; Pain; Pins and needles; Stoma; Recurrence; Diabetes; Sexual dysfunction |
| **C. PSYCHOLOGICAL (9 themes)** | |
| **C.1. COPING WITH CANCER AND A NEW REALITY (2 themes)** | |
| Personal disposition [72] | Optimism; Stoicism |
| Paying a price for survival [74] | The need to use pads and a feeling of being a child with a diaper when having a  drink at a party was balanced by the relief of being rid of the cancer. |
| **C.2. CANCER-RELATED ANXIETY & DISTRESS (3 themes)** | |
| *Onset and nature [72] | Anxiety; Cognitive impairment; Depression; Fear of recurrence; Sleep disturbance |
| Changes in outlook [73] | “You have to take yourself anew don’t you? You definitely do not feel in any shape or form the sort of person who you were before. You’ve been chucked up in the air and you come back down again and everything’s different, it feels to me.” |
| Embodied reminders [73] | Even if participants could reconcile their fear of recurrence, they still had physical reminders of their treatment. Some participants described tattoos and burn marks from radiotherapy; others complained of weight gain resulting from hormone therapy. In addition, all of the participants had lost either part, or the whole, of one or both breasts. |
| **C.3. BODY IMAGE (1 theme)** | |
| *Onset and nature [72] | Body image issues |
| **C.4. FEAR OF RECURRENCE (3 themes)** | |
| Fear of recurrence [73] | One of the reasons why participants seemed to be in an ambiguous state was that despite having completed treatment, they were aware that the cancer could potentially return. |
| Living with death lurking around the corner [74] | However, during and after contact with the hospital, and in other situations reminding them about their cancer, their thoughts about death grew as strong as when they were first diagnosed |
| *Onset and nature [72] | Fear of recurrence |
| **D. SOCIAL (3 themes)** | |
| **D.1. SOCIAL RELATIONSHIPS (1 theme)** | |
| Relationships [73] | While participants strove to sustain the pretence of ‘normality’ in social situations, this was not achievable in intimate situations […] |
| **D.2. SOCIAL SUPPORT AND STIGMA (1 theme)** | |
| Peer comparisons [72] | Comparison of patient late effects experience in relation to other survivors they know, had read or heard about |
| **D.3. WORK (1 theme)** | |
| Impact of late effects [72] | Working status (employment, reduction of working hours, reduced ability to work, financial impact); Impact on activities of daily living; working status (employment, reduction of working hours, reduced ability to work, financial impact) |
| **E. LIFE DISRUPTION (3 themes)** | |
| Biographical disruption and liminality [73] | "It just shatters you completely. Just everything, everything alters. Your whole life sort of thing." |
| Feeling sidestepped [74] | The men felt that the disease was not prioritised by society or the health care system. |
| Sense making [72] | "intra-individual process of trying to understand the cause of their initial cancer and subsequent late effects and experienced difficulty untangling the cause of late effects in relation to other illnesses, family history and the effects of ageing" |

*Themes distributed in two or more categories according to the content of the subthemes and verbatims.

**Supplementary table 6.** Results of thematic analysis of studies focused on working situation.

| **A. CLINICAL MANAGEMENT (1 theme)** | |
| --- | --- |
| **A.1. INFORMATION AND COMMUNICATION (1 theme)** | |
| Cancer treatment and late effects [77] | Interviewees described how it was important to have information about treatment and, especially, the time it would take. Several underlined the importance of predictability and participation in when and how the treatment was supposed to be executed. This was important for their overall well-being but also of particular importance for practicalities related to managing their business. |
| **B. PSYCHOLOGICAL (2 themes)** | |
| Shame [77] | “I felt like a second-class item—the weak chicken of the flock that the fox takes. For me, cancer was a hag disease. Young and ft me, I was in fine shape! To me, those who got breast cancer were some heaps of fat sitting inside while smoking” |
| To be a minus [75] | Reduced confidence in work life “(I)... am sort of a minus) |
| **C. SOCIAL (10 themes)** | |
| **C.1. SOCIAL SUPPORT AND STIGMA (2 theme)** | |
| Those who are closest have a lot to say – hard without [75] | Support from relatives (“It’s clear that those who are closest to me; wife, parents. It means an awful lot to say that you have a support system around you. You need to have that... If not ... it becomes terribly hard”) |
| Support [77] | “If it hadn’t been for my husband this business wouldn’t have existed” […] “My husband had to take over everything with the kids when I got treatment. Deliver the kids in kindergarten and also to milk the cows. We got a substitute worker but my father also helped with feeding the cows. When I was at home (from the hospital) during the week-end I could take my share” |
| **C.2. WORK (8 themes)** | |
| Decision making for returning to work [76] | (1) uncertainty; (2) facilitators  1)It returning to work was a sign of healing, and I proved myself. I decided to work for a few months and then to get retired, but I didn't get retired since I felt better;  2) My breasts were removed. After that, I became anxious about my physical appearance. I wondered about how my colleagues would treat me about it |
| Difficulties in work life [76] | (1) burden of symptoms; (2) inability to modify lifestyle; (3) negative attitudes of employers and colleagues "1) One difficulty I experienced at work was the effort I had to make to prevent swelling in my arm likely to be due to removal of the lymph nodes. I also feel weak and tired I experience a great difference now compared with the time before the cancer; 2) The doctor told me to go for a walk. I can't do it"; 3) I felt good when I returned to work, but many people have heard about my disease, and I got a bit bored with having to tell the things again and again" |
| Sources of motivation for continuation of work life [76] | (1) familial support; (2) having a supportive workplace atmosphere; (3) what cancer has taught 1) My family says that I was more withdrawn and quieter before returning to work, but that I became more active and took care of myself better. This has a positive influence on me ; 2)I was allowed to have some flexibility in working hours. Sometimes I can be late for work, and they (employers) show tolerance for it. When I want to leave, no one objects to it; 3)I used to be reserved. I used to keep silent not to make my boss upset. Now I want to tell what I like without hurting people. I don't want to get distressed anymore because I have one life and want to live it happily and peacefully |
| Benefits of returning to work [76] | (1) psychological improvement; 2) socialization 1) It improves one's mood. It relaxes me psychologically; 2) You become involved in life. You learn things from people around. You become socialized more |
| Entrepreneurship and engagement [77] | “I think if you love your business, you will enjoy working. The job will embrace you and you forget everything else. Work is a big part of me and important to get me going. I work everywhere…. I don’t do this because of the money. Of course, I need housing and bread, but if I had won the lottery I would still have worked. The work engagement is such a big part of me” |
| Business related worries [77] | “I have no time to be sick! Will I lose customers? What can I do? Do I have to shut down my business?” |
| Back at work 100% after a couple of months [75] | Expectations of RTW “Then I was a little like; everything like before? Then I’ll be back at work 100%, after a couple, three months |
| Adjustments of work tasks is everything [75] | Adjustments “To get the adjustments in (...) really is everything (...) in relation to the job” |

*Themes distributed in two or more categories according to the content of the subthemes and verbatims.

**Supplementary table 7.** Results of thematic analysis of studies focused on psychological distress.

| **A. CLINICAL MANAGEMENT (1 theme)** | |
| --- | --- |
| **A.1. RELATIONSHIP AND SUPPORT FROM PROFESSIONALS (1 theme)** | |
| Unmet support needs [80] | Information support; Psychological support; Health care support; Social support |
| **B. SYMPTOMS & PHYSICAL FUNCTION (2 themes)** | |
| My brain is not functioning [79] | Concentration; Memory; Keeping up with conversation |
| My body hurts [79] | Fatigue; Pain; Headaches; Nausea |
| **C. PSYCHOLOGICAL FUNCTION (4 themes)** | |
| **C.1. CANCER-RELATED ANXIETY & DISTRESS (4 themes)** | |
| Perceptions of loss [78] | Perceptions regarding loss of function; Perceptions regarding loss of self; Perceptions regarding loss of connection; Perceptions regarding loss of control; Psychological vulnerability: exacerbating factors |
| I don’t feel like myself [79] | Irritable; Lacking motivation; Avoidance; Loss of interest; Frustration; Guilt about tiredness |
| Sources of psychological distress [80] | Physical symptoms; Emotional problems; Relationship problems |
| Maladaptive strategies for coping with distress [78] | Concealment of distress; Avoidance of help seeking; Withdrawal (social/activity) |
| **D. SOCIAL FUNTION (2 themes)** | |
| **D.1. SOCIAL RELATIONSHIPS (1 theme)** | |
| It’s more than just not sharing a bed [79] | Sleeping separately; Missing out on conversations; Partner irritation; Different bedtimes |
| **D.2. SOCIAL SUPPORT AND STIGMA (1 theme)** | |
| Barriers to support [80] | Negative experiences; Prejudices; Avoidance |
| **E. LIFE DISRUPTION (2 themes)** | |
| Worry [79] | Racing mind; Pre-occupation with sleep; Pressure to get back to normal |
| Planning life around something uncontrollable [79] | Withdrawn/Isolated; Not making plans; Giving up work |

*Themes distributed in two or more categories according to the content of the subthemes and verbatims.
